# Spatiotemporal Encapsulation of Tandem Enzymes in Hierarchical Metal–Organic Frameworks for Cofactor‐Dependent Photoenzymatic CO_2_ Conversion

**DOI:** 10.1002/advs.202410024

**Published:** 2024-11-08

**Authors:** Yan Li, Jieqiong Wang, Xiaoqian Shi, Xiaoxuan Yu, Shuangjiang Yu, Junqiu Liu, Hongcheng Sun

**Affiliations:** ^1^ Key Laboratory of Organosilicon Chemistry and Material Technology Ministry of Education College of Material Chemistry and Chemical Engineering Hangzhou Normal University Hangzhou 311121 China

**Keywords:** CO_2_ fixation, metal–organic frameworks (MOFs), photoenzymatic catalysis, spatiotemporal encapsulation, tandem enzymes

## Abstract

The photo‐enzyme coupling system (PECS) holds immense potential in “green” biomanufacturing, encompassing the realms of pharmaceuticals, fuels, and carbon sequestration. Nevertheless, the intricate nature of enzymes' structures significantly impedes the seamless integration of multiple enzymes in a precise, tandem fashion, with exact control over their distribution, posing a formidable challenge. Herein, it has devised a mesoporous *csq*‐type metal organic framework (Zr‐MOF) from *meso*‐tetrakis‐(4‐((phenyl)ethynyl)benzoate)porphyrin (Por‐PTP) and Zr_6_(μ_3_‐O)_4_(μ_3_‐OH)_4_(OH)_4_(H_2_O)_4_) nodes (Zr_6_ clusters), featuring intricate hierarchical hexagonal (5.8 nm) and triangular (2.9 nm) channels, enabling the simultaneous encapsulation of Formate dehydrogenase from *Candida boidinii* (*Cb*FDH) and ferredoxin‐NADP+ reductase (FNR) via a spatiotemporally controlled strategy for cofactor‐dependent photoenzymatic carbon dioxide (CO_2_) conversion. Upon illumination, photoexcited electrons originating from the Zr‐MOF frameworks migrate to the adjacent FNR for cofactor NADH regeneration, which is then harnessed by proximal *Cb*FDH for CO_2_ fixation. Concurrently, the resulting holes are neutralized by AA for system recovery. The results demonstrated the confinement of tandem enzymes within MOF channels significantly enhanced the performance of multi‐enzyme cascade pathways as well as augmenting the local NAD^+^/NADH, which leading to a further improvement in the efficiency of tandem biocatalytic formic acid generation (55 mm) from CO_2_. Crucially, the photo‐enzyme‐coupled factories exhibited remarkable stability alongside exceptional recyclability, attributed to the preservation of MOF skeletons.

## Graphical abstract

The semi‐artificial photocatalyst‐multienzyme‐coupled catalytic system is constructed through spatiotemporal encapsulation of tandem enzymes for carbon dioxide (CO_2_) photoreduction is presented. Enzymes are encapsulated in a tandem manner with precise control of the distribution into metal‐organic frameworks. The hybrid system facilitates photocatalytic cofactor NADH regeneration and converts CO_2_ into formic acid with high efficiency.

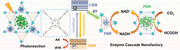

## 1 Introduction

In recent decades, the mounting energy crisis and pervasive environmental pollution have spurred the rapid advancement of technologies aimed at harnessing renewable and sustainable energy sources, including biomass, wind power, and solar energy.^[^ ^1^ ^]^ Natural photosynthesis, ubiquitously observed in plants, algae, and even bacteria, constitutes one of the most elegant and potent pathways for environmentally benign, renewable transformation of naturally abundant carbon dioxide (CO_2_) into valuable chemicals (biomass).^[^ ^2^, ^3^ ^]^ During this process, the photoexcited electrons are transferred from pigment‐protein complexes via electron‐transport chains to the ferredoxin‐NADP^+^ reductase (FNR) for regeneration of reduced nicotinamide adenine dinucleotide phosphate (NADPH), which could be efficiently utilized by tandem enzymes for generation of carbohydrates during “dark reaction” pathways like the Calvin cycle, the Hatch–Slack pathway and so on.^[^ ^4^, ^5^ ^]^ The photosynthesis process exquisitely harnesses the selectivity of enzymes and unique chemical transformations, accomplishing this in a biologically benign and environmentally friendly manner.^[^ ^6^, ^7^ ^]^ Inspired by natural photosynthesis systems, semi‐artificial photosynthesis that harmoniously integrating photochemical processes with enzymatic reactions has emerged as the most promising and efficient pathway toward harnessing light energy for the resourceful utilization of CO_2_.^[^ ^8^, ^9^ ^]^

Formate dehydrogenase from *Candida boidinii* (*Cb*FDH) is a nicotinamide adenine dinucleotide (NAD(P)^+^/NAD(P)H)‐a dependent enzyme with dimensions ≈4.0 nm × 6.0 nm × 11 nm, which can catalyze the dehydrogenation of formic acid to CO_2_ or its reverse process in the presence of NAD(P)^+^/NAD(P)H cofactors.^[^ ^10^ ^]^ In recent years, *Cb*FDH has been successfully harnessed for developing semi‐artificial photo‐enzyme‐coupled catalytic systems (PECSs) for CO_2_ fixation, which encompass vital components such as sacrificial agents, photosensitive components, NAD(P)^+^ cofactor, electron mediator, and the biocatalytic enzyme *Cb*FDH.^[^ ^11^, ^12^ ^]^ In these PECSs, titanium dioxide (TiO_2_),^[^ ^13^, ^14^ ^]^ quantum dots,^[^ ^15^ ^]^ graphite phase carbon nitride (g‐C_3_N_4_),^[^ ^16^ ^]^ fluorochromes^[^ ^17^ ^]^ and organic frameworks,^[^ ^12^, ^18^ ^]^ have been extensively utilized as photosensitizer for improved light harvesting and exciton dissociation. The rhodium‐based complex Cp*Rh(bpy)Cl (Cp* = pentamethylcyclodienyl; bpy = bipyridine), functions as an electron mediator, frequently utilized to resolve the energy level disparity between an excited‐state photosensitizer and redox cofactor. In addition, the FNR enzyme serves as an efficient electron mediator in biological systems, specializing in the regeneration of NAD(P)H from NAD(P)^+^.^[^ ^15^, ^19^ ^]^ For example, Yehezkeli and Brown have presented protein/semiconductor biohybrids that were coupled with FNR mediator for the photocatalytic regeneration of NADPH cofactor.^[^ ^15^, ^20^ ^]^ However, the inherent issues of enzyme degradation and significant leaching that frequently occur under operational conditions, achieving sustained long‐term enzymatic activities can pose a formidable challenge.

Metal‐organic framework (MOF),^[^ ^21^, ^22^ ^]^ constructed via intricate metal coordination interactions between metal nodes and organic ligands, represents an ultrahigh porosity crystalline material with a hierarchical porous structure. Such hierarchical porous structure renders it exceptionally versatile, enabling the customization of diverse structures and functionalities tailored specifically for targeted applications.^[^ ^23^, ^24^, ^25^, ^26^ ^]^ MOFs have emerged as versatile biomimetic exoskeletons, showcasing their capability to encapsulate a broad spectrum of biological entities, extending beyond enzymes alone, thereby enhancing their structural integrity and promoting recyclability.^[^ ^27^ ^]^ The merits of harnessing PECSs for CO_2_ fixation through MOF encapsulation strategy are outlined as follows: 1) Integration of photosensitizing units into a mesoporous MOF scaffold can effectively organize the chromophores for enhanced energy transfer efficiency;^[^ ^28^ ^]^ 2) The hierarchical mesoporous structures derived from MOFs, possessing dimension comparable to *Cb*FDH would facilitate the infiltration of enzyme into the frameworks, thereby preserving both the enzyme structure and functionality;^[^ ^27^ ^]^ 3) The ultrahigh porosity and tunable nature of MOF would significantly enhance CO_2_ capture and expedite the access to enzyme center; and 4) The *Cb*FDH in a confined microenvironment has the potential to minimize the diffusion process of the regenerated substrate toward the enzyme, ultimately enhancing the efficiency of light‐driven CO_2_ fixation. For example, Liu group incorporated Cp*Rh complex and FDH into MOF MIL‐125‐NH_2_ for facilitating photocatalytic NADH regeneration and photoenzymatic catalysis of CO_2_ conversion.^[^ ^29^, ^30^ ^]^ Li and Farha reported an integrated *Cb*FDH@Rh‐NU‐1006 system for high‐efficiency CO_2_ reduction with recyclable cofactor through enzyme encapsulation strategy with NU‐1006, which features an extended *csq‐net* topology with the mesoporous hexagonal channels and the triangular channels interconnected with the orthogonal windows.^[^ ^10^, ^31^ ^]^

Compared with natural biological photosynthetic systems, multistep enzyme cascades are typically utilized to generate a diverse array of chemical commodities, thereby providing a time‐efficient and cost‐effective alternative to single‐step reactions.^[^ ^32^ ^]^ At present, it is still difficult to efficiently couple both two different enzymes into a tandem nanofactory with precise control of the distribution, due to their competitive incorporation with MOF traps.^[^ ^33^, ^34^ ^]^ It is also a rare occurrence in developing PECSs for CO_2_ fixation. Predictably, the rationally designed architectures of *csq‐net* topological MOF, featuring hierarchical channel structures, could potentially facilitate the spatially partitioned co‐immobilization of tandem enzymes within distinct channels. Simultaneously, the excessive orthogonal windows that interconnect these channels ensure accessibility for efficient mass transport during the photocatalysis process.

**Scheme 1:**
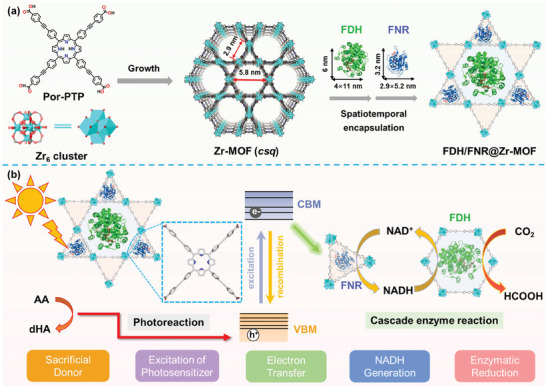
a) Schematic representation of solvothermal synthesis of Zr‐MOF from Por‐PTP and Zr_6_ cluster (left). Stepwise immobilization of *Cb*FDH and FNR in the mesopores of Zr‐MOF to obtain FDH/FNR@Zr‐MOF. b) Illustration of photocatalytic regeneration of NADH for formic acid production from CO_2_ over FDH/FNR@Zr‐MOF.

Herein, we tailed a PECS with precisely arranged multiple enzyme cascades within MOFs for cofactor‐dependent photoenzymatic CO_2_ conversion through an innovative spatiotemporal encapsulating strategy. *Meso*‐tetrakis‐(4‐((phenyl)ethynyl)benzoate)porphyrin (Por‐PTP) and zirconium oxocluster (Zr_6_ cluster) node were developed as building blocks to construct a water‐stable mesoporous MOF (denoted as Zr‐MOF) with *csq‐net* topology for light harvesting and enzyme encapsulation. By virtue of their substantial pore dimensions and intricate hierarchical topological structures, the *Cb*FDH and FNR enzymes were spatiotemporally sequestered within larger hexagonal mesopores (5.8 nm width) and moderate triangular mesopores (2.9 nm width) respectively through a meticulous, size‐guided, step‐by‐step infiltration process (**Scheme** **1**a).^[^ ^27^ ^]^ What's more, these parallelly arranged mesopores were interconnected by the orthogonal diamond windows (2.9 nm width) for substance transportation during the reaction processes. Upon illumination, the photogenerated electrons within the Zr‐MOF framework were efficiently channeled to the encapsulated FNR center to facilitate the reduction of NAD^+^ into NADH, while the accompanying photogenerated holes were efficiently consumed by the sacrificial agent ascorbic acid (AA) for ensuring electric neutrality within the system.^[^ ^15^, ^35^ ^]^ Afterward, the locally generated NADH traversed the orthogonal windows to access the adjacent encapsulated *Cb*FDH for facilitating the reduction of CO_2_ into formic acid (Scheme 1b). The precise distribution of tandem enzymes within the Zr‐MOF channels facilitated both the local NAD^+^/NADH concentration and the substance transformation, thereby significantly improving the final CO_2_ conversion efficiency. Besides, the MOF exoskeletons have also preserved structural robustness and recyclability, thereby paving a new pathway for multiple‐enzyme‐cascade PECSs for developing green bio‐manufacturing factories.

## 2 Results and Discussion

### 2.1 Preparation and Characterization of Mesoporous Zr‐MOF

To achieve spatially partitioned co‐immobilization of FNR and *Cb*FDH enzymes with different dimensions, it is necessary to construct a water‐stable, hierarchical mesoporous MOF structure with mesopores well‐matched with different enzymes. Recent reports highlighted that the ***ftw*** topological NU‐1104 MOF, which was constructed by *meso*‐tetrakis‐(4‐((phenyl)ethynyl)benzoate)porphyrin (Por‐PTP) and zirconium oxoclusters (Zr_6_(μ_3_‐O)_4_(μ_3_‐OH)_4_(OH)_4_(H_2_O)_4_, Zr_6_ clusters)) with the modulation of benzoic acid, featured large cubic cages with a pore diameter ≈2.9 nm.^[^ ^36^ ^]^ It was predicted that the formation of a hierarchical ***csq***‐net topological structure could feature parallelly arranged hexagonal channels (≈5.8 nm pore width) and triangular channels (≈2.9 nm pore width), which would align well with the dimensions of *Cb*FDH and FNR enzymes, respectively. Intriguingly, the co‐immobilization of both tandem FNR and *Cb*FDH not only facilitates the diffusion of regenerated NADH cofactor and substrates via a narrower tetragonal channel interstice but also maintains their structural integrity and catalytic prowess under adverse conditions for enhancing photo enzymatic catalytic efficiency.

**Figure 1:**
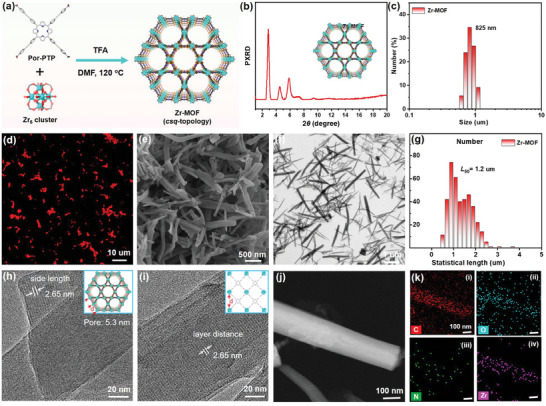
a) Solvothermal synthesis of Zr‐MOF from Por‐PTP and Zr_6_ cluster. b) The power X‐ray diffraction (PXRD) of Zr‐MOF. c) The hydrodynamic diameter of Zr‐MOF. d) CLSM, e) SEM and f) TEM images of Zr‐MOF. g) Statistical distribution of the length of Zr‐MOF nanorods in image (f). h,i) High‐resolution TEM images of Zr‐MOF. j) Dark‐field high‐resolution TEM and k) corresponding EDX elemental mappings of Zr‐MOF nanorods; C (i, red), O (ii, cyan), N (iii, green) and Zr (iv, pink).

Herein, we synthesized a tetracarboxylate porphyrinic linker, designated as Por‐PTP, via meticulous stepwise cyclization processes of home‐made *tert*‐butyl 4‐((4‐formylphenyl)ethynyl)benzoate monomer (tBut‐BzH), followed by an ester hydrolysis reaction (Scheme S1, Supporting Information), according to the modified literature.^[^ ^36^ ^]^ The nuclear magnetic resonance spectroscopy (^1^H NMR) and mass spectrometry techniques including liquid chromatography‐mass spectrometry (LC‐MS) and matrix‐assisted laser desorption/ionization time‐of‐flight (MALDI‐TOF) were utilized to validate and confirm the molecular structures of synthesized intermediates throughout the reaction processes (Figures S1–S6, Supporting Information). As depicted in Figures S7 and S8 (Supporting Information), the chemical shift in ^1^H NMR and mass‐to‐charge (m/z) ratio outcomes by MALDI‐TOF spectroscopy align favorably with the theoretical predictions of Por‐PTP, thus confirming the successful synthesis of the Por‐PTP ligand. As schematically illustrated in **Figure** **1**a, ***csq***‐net topological MOF was further constructed through solvothermal reaction of tetracarboxylate Por‐PTP ligand and freshly made Zr_6_ clusters (Zr_6_(μ_3_‐O)_4_(μ_3_‐OH)_4_(OH)_4_(H_2_O)_4_) at 120 °C for 6 h with appropriate amount of trifluoroacetic acid (TFA) and N, N‐dimethylformamide (DMF) as modulator and solvent, respectively.^[^ ^37^, ^38^ ^]^

Fourier transform infrared (FT‐IR) spectroscopy was initially utilized to demonstrate the formation of MOF structures. As shown in Figure S9 (Supporting Information), the Por‐PTP features strong infrared peaks at 1696 and 1204 cm^−1^ for the stretching vibrations of C═O and COOH, respectively. After the formation of the Zr‐MOF, these peaks underwent a significant reduction accompanied by the emergence of new peaks at 1658 cm^−1^, signifying the coordination between COOH groups and Zr_6_ clusters. As depicted in Figure 1b, the powder X‐ray diffraction (PXRD) analysis clearly indicates that the Zr‐MOF exhibited pronounced diffraction peaks (2θ) at 2.9°, 4.5° and 5.9°, which was quite in accordance with the previous reported ***csq***‐topological structures.^[^ ^10^ ^]^ Furthermore, the dynamic light scattering (DLS) analysis presented in Figure 1c revealed the hydrodynamic diameter of the Zr‐MOF was ≈825 nm.

Notably, owing to the porphyrin‐based skeletal structure, the Zr‐MOF features rod‐shaped structures with robust red fluorescence, as evidenced by the confocal laser scanning microscope (CLSM) measurements depicted in Figure 1d. The morphological details of the Zr‐MOF were further conducted utilizing scanning electron microscope (SEM) and transmission electron microscopy (TEM) measurements. As depicted in Figure 1e,f, distinctive, needle‐like single crystals with microscale dimensions were discernibly observed in both SEM and TEM images. Upon statistically analyzing the length distribution in Figure 1f, the Zr‐MOF features distinct hexagonal cylinder‐shaped crystals with lengths predominantly ranging from 0.7 to 2.1 µm and a median length (*L*_50_) of ≈1.2 µm (Figure 1g), which aligned well with the results obtained from the DLS measurement. Besides, high magnification TEM measurement was utilized to reinforce the specifics pertaining to the Zr‐MOF structures. A parallelly arranged lattice fringe with a fringe spacing ≈2.65 nm was observed in Figure 1h,i. It can be measured that the size of the hexagonal mesopore would be 5.3 nm from TEM results, which exhibits a precise correlation with the theoretical structures (5.8 nm). TEM image (Figure 1j) and its corresponding energy‐dispersive X‐ray spectroscopic (EDX) mappings in (Figure 1k) indicated their hexagonal cylinder‐shaped crystals with the uniformly dispersed C (i, red), O (ii, cyan), N (iii, green) and Zr (iv, pink) elements throughout the entire structures.

### 2.2 Spatiotemporal Immobilization of C*b*FDH and FNR in Zr‐MOF

As is well‐known, the immobilization of enzymes within MOFs via postsynthetic infiltration approach is typically dictated by the pore dimensions of MOF structures. Efficiently integrating two distinct enzymes into a tandem nanofactory while maintaining precise control over their distribution poses a challenge due to their competitive incorporation with the MOF traps.^[^ ^27^ ^]^ Hierarchical porous frameworks of the Zr‐MOFs offer the potential to synchronously encapsulate different enzymes with exceptional loading efficiency.^[^ ^33^ ^]^ An initial examination suggested that *Cb*FDH and FNR could potentially accommodate within the hexagonal and triangular mesopores of the Zr‐MOFs, respectively.^[^ ^10^ ^]^ To validate this concept, the two enzymes were successfully expressed and integrated into the Zr‐MOFs through a precise spatiotemporal immobilization approach. The expression of *Cb*FDH and FNR enzymes was accomplished utilizing *Escherichia coli* expression system, involving the introduction of *Cb*FDH‐Pet28a(+) and FNR‐Pet28a(+) plasmids, respectively, into BL21(DE3) competent cells (Figures S10 and S11, Supporting Information). Subsequently, the expressed enzymes were further purified through a multi‐step process involving Ni‐NTA affinity column, desalting column, and ultrafiltration tubes, yielding highly purified enzymes. The sodium dodecyl sulfate‐polyacrylamide gel electrophoresis (SDS‐PAGE) in Figure S12 (Supporting Information) indicated the successful expression and purification of *Cb*FDH and FNR enzymes.

**Figure 2:**
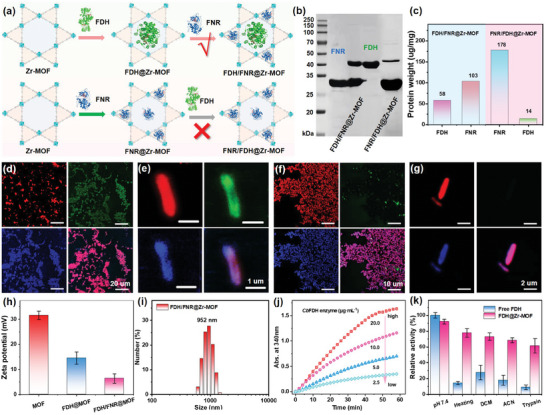
a) Graphic representation of the results of the spatiotemporal immobilization of *Cb*FDH and FNR with different orders. b) SDS‐PAGE analysis of FDH/FNR@Zr‐MOF and FNR/FDH@Zr‐MOF (analyzed after the dissolution of MOF). c) Bicinchoninic acid (BCA) analysis of FDH/FNR@Zr‐MOF and FNR/FDH@Zr‐MOF for quantification. Colocalization staining of d,e) FDH/FNR@Zr‐MOF and f,g) FNR/FDH@Zr‐MOF from CLSM measurements. CbFDH was labeled with FITC (green), and FNR was labeled with coumarin (blue). h) Zeta‐potential and i) size distribution of FDH/FNR@Zr‐MOF from DLS measurements. j) Kinetic curve of *Cb*FDH enzyme for HCOOH oxidation versus *Cb*FDH content (2.5, 5.0, 10.0, and 20.0 µg mL^−1^) in the presence of NAD^+^. k) Retained enzymatic activity of free FDH (cyan) and FDH@Zr‐MOF (pink) before (pH 7.4) and after treatment with elevated temperature (50 °C), organic solvents (DCM, ACN) and trypsin.

After that, two different sequences of enzyme infiltration were employed to evaluate the loading efficacy of each individual enzyme. For the sequence of FDH/FNR@Zr‐MOF (*Cb*FDH first, then FNR), the Zr‐MOFs were incubated with large *Cb*FDH to occupy the hexagonal channels of Zr‐MOFs, subsequently incubation with FNR to load the smaller channels (**Figure** **2**a, up).^[^ ^39^ ^]^ For the adverse sequence of FNR/FDH@Zr‐MOF, the Zr‐MOFs were used to encapsulate small FNR first and then for *Cb*FDH encapsulation (Figure 2a, down). The encapsulation of *Cb*FDH or FNR was achieved by immersing Zr‐MOF into an excessive amount of enzyme solution until saturation, then retrieved and rigorously washed with buffer three times to remove any shakily bonded enzymes. The SDS‐PAGE and the bicinchoninic acid (BCA) assays were employed to confirm the loading amounts of *Cb*FDH and FNR. As depicted in Figure 2b,c, the FDH/FNR@Zr‐MOF system displayed comparable electrophoresis strips for both *Cb*FDH and FNR enzymes in SDS‐PAGE analysis, whereas higher FNR uptake and lower *Cb*FDH encapsulation were found for the reversed ordered FNR/FDH@Zr‐MOF system. The BCA analysis disclosed the packaging capabilities of FNR and *Cb*FDH enzymes within the FDH/FNR@Zr‐MOF system were 103 and 58 µg mg^−1^ respectively, whereas these values were 178 and 14 µg mg^−1^ respectively for the FNR/FDH@Zr‐MOF system. These findings suggested that *Cb*FDH and FNR enzymes for the FDH/FNR@Zr‐MOF system potentially occupy the hexagonal channels and triangular channels of Zr‐MOFs, respectively. However, smaller FNR enzymes will infiltrate into the hexagonal and triangular channels concurrently if reversing the order to FNR→ *Cb*FDH (FNR/FDH@Zr‐MOF), thereby significantly impeding the efficient incorporation of CbFDH.

Additionally, CLSM was utilized to examine the colocalization of the Zr‐MOF structure of two different enzymes in detail. Because the Zr‐MOF exhibited a robust red fluorescence emanating from its porphyrin subunit. In this study, we performed a colocalization staining analysis utilizing a green fluorescence probe, fluorescein isothiocyanate isomer (FITC), and a blue fluorescence probe, coumarin‐6‐sulfonyl chloride (CSCl), to distinctly label *Cb*FDH and FNR enzymes, respectively. As depicted in Figure 2d,f, the FDH/FNR@Zr‐MOF system exhibited exceptionally robust tricolor fluorescence (red, green, and blue) with exceptional overlap, whereas only red and blue fluorescence could be clearly found for the FNR/FDH@Zr‐MOF system. If further localizing into individual Zr‐MOF nanorod, both *Cb*FDH and FNR were uniformly dispersed in the whole Zr‐MOF structure in FDH/FNR@Zr‐MOF but only FNR overlapped in FNR/FDH@Zr‐MOF (Figure 2e,g), indicating the co‐encapsulation of two enzymes rather than the random absorption in FDH/FNR@Zr‐MOF. These results demonstrated that the spatiotemporal immobilization with the order large *Cb*FDH→small FNR can synchronously enhance the encapsulation capacity of both *Cb*FDH and FNR through spatially partitioned immobilization, thereby enabling the development of multi enzymatic “light factory” for photocatalytic CO_2_ activation and resource utilization.

Besides, the thermogravimetric analysis (TGA) indicated the successful encapsulation of enzymes into Zr‐MOF frameworks (Figure S13, Supporting Information). The density functional theory (DFT) pore size distribution analysis also indicated that the pore volume corresponding to the triangular channels and hexagonal channels of Zr‐MOF dropped dramatically after enzymes encapsulation (Figure S14, Supporting Information). The zeta‐potential of Zr‐MOFs gradually decreased from 31.5 to 7.8 mV after stepwise encapsulation of *Cb*FDH and FNR enzymes (Figure 2h), due to their intrinsic negative charges of enzymes. Meanwhile, the hydrodynamic size of FDH/FNR@Zr‐MOF exhibited a comparable size (952 nm) with that of individual Zr‐MOFs (825 nm) from the DLS measurement (Figure 2i). The *Cb*FDH exhibited a characteristic catalytic kinetic profile, showcasing an enzyme activity of ≈31.8 µmol h^−1^ g^−1^, as derived from the *Cb*FDH activity assay (Figure 2j). Moreover, the thiazolyl blue tetrazolium bromide (MTT) colorimetric assay validated the generation of NADH, as it can reduce the MTT to a water‐insoluble blue‐purple crystalline formazan with enhanced absorption within the wavelength range of 480–630 nm (Figure S15, Supporting Information). The spatial confinement of tandem enzymes within MOFs not only preserves their inherent catalytic performance through augmenting metabolic pathways but also ensures their structural robustness, even under stringent environmental conditions. It was found more than 90% activity recovered for encapsulated *Cb*FDH (FDH@Zr‐MOF) versus free FDH at pH 7.4. Not only that, the encapsulated *Cb*FDH retained higher catalytic activity than free *Cb*FDH after enduring rigorous treatment conditions, encompassing elevated temperature, exposure to organic solvents, or even the presence of proteases. It was found that the system activity could be recovered to over 60% in the case of FDH@Zr‐MOF upon exposure to elevated temperatures (50 °C), dichloromethane (DCM), acetonitrile (ACN), or trypsin, respectively (as depicted in Figure 2k). In contrast, only 20% of activity was restored for free *Cb*FDH. This finding underscores the potential of these materials in developing photo‐enzyme‐coupled CO_2_ fixation systems.

### 2.3 Optical and Photoelectric Properties

**Figure 3:**
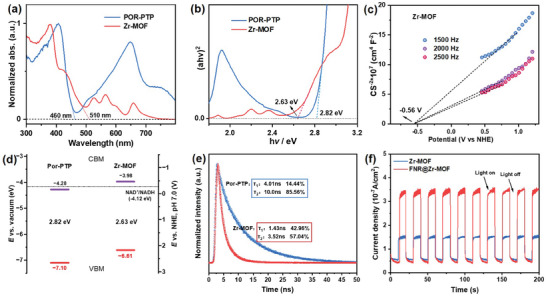
a) The solid‐state ultraviolet–visible diffuse reflectance (UV–vis–DRS) spectra and b) the corresponding *Tauc* plot of Por‐PTP (blue) and Zr‐MOF (red). c) Mott–Schottky (M−S) plots of Zr‐MOF at 1500, 2000 and 2500 Hz. d) Experimental estimated band structure diagrams, e) The time‐resolved photoluminescence of Por‐PTP (blue) and Zr‐MOF (red). f) Photocurrent response curves of Zr‐MOF and FNR@Zr‐MOF.

To demonstrate their proficiency in photocatalytic NADH generation, a comprehensive investigation of the photophysical properties of Zr‐MOF was undertaken, employing a diverse array of optical and photoelectronic techniques. The Por‐PTP molecule exhibited a strong absorption peak at 421 nm from UV–vis spectra (Figure S16, Supporting Information) and robust emission peaks at 656 nm and 722 nm from fluorescence spectra (Figure S17, Supporting Information). Solid state ultraviolet–visible diffuse reflectance spectra (UV–vis DRS, **Figure** **3**a) revealed the Por‐PTP exhibited a broad UV–vis absorption band edged at 460 nm and a strong Q band between 460 and 800 nm because of the strong π–π stacking. After coordination into Zr‐MOF, the edge of the absorption band redshift to 510 nm with Q band decreased dramatically. Such an expanded absorption band contributes significantly to enhancing the capability for light absorption during photocatalysis. The optical energy band gaps (*E*_g_) of the Zr‐MOF, as determined by *Tauc* plots, were ≈2.63 eV, narrower than the 2.82 eV gap of Por‐PTP ligand, suggesting a more feasible charge separation process under visible light absorption, as depicted in Figure 3b.

The Mott–Schottky (M−S) measurements indicated Zr‐MOF and Por‐PTP exhibited positive slopes, as depicted in Figure 3c and Figure S18 (Supporting Information), indicating the n‐type semiconductor behaviors. The potentials (***E*** (NHE)) relative to the normal hydrogen electrode (NHE, pH 7.0) were measured to be ≈−0.26 and −0.56 V for Por‐PTP and Zr‐MOF, respectively, from experimentally measured potentials (***E*** (SSC)) versus Ag/AgCl electrode (SSC, [Cl^−^] = 3.5 m) using the following equation: ***E *** (NHE) = ***E*** (SSC) + 0.20 (V), where 0.20 V is the electrode potential of the SSC electrode in a 3.5 m potassium chloride solution. The ***E*** (NHE) values were converted to −4.18 and −3.88 eV versus vacuum levels (−4.44 eV for NHE) for Por‐PTP and Zr‐MOF, respectively, according to the equation: ***E *** (vac) = −4.44 − ***E*** (NHE) (eV). Considering the ***E*** (NHE) is typically positioned at a more positive value (≈0.1 eV) than the energy levels of the conduction band minimum (CBM) for n‐type semiconductors, owing to an excess of electrons, the CBM versus vacuum level was estimated to be −4.28 eV for Por‐PTP and −3.98 eV for Zr‐MOF, respectively. The energy levels of the valence band maximum (VBM) were estimated to be −7.10 eV for Por‐PTP and ‐6.61 eV for Zr‐MOF, respectively, and the derived band structures over Zr‐MOF were presented in Figure 3d. Compared to the reduction potential of NAD^+^/NADH (−4.12 eV), the CBM of Zr‐MOF exhibited a more negative value, thereby suggesting the viability of Zr‐MOF in medicating the photoreduction of NAD^+^ to NADH.

Meanwhile, the time‐resolved fluorescence decay analysis revealed a shorter relaxation lifetime for Zr‐MOF (*τ*_1_ = 1.43 ns; *τ*_2_ *=* 3.52 ns) compared to Por‐PTP (*τ*_1_ = 4.01 ns; *τ*_2_ *=* 10.0 ns), suggesting that the assembly of Por‐PTP linkers into the MOF framework facilitates the electron transfer efficiency (Figure 3e). The cyclic voltammetry (CV) measurement in Figure S19 (Supporting Information) revealed Zr‐MOF had a more negative reduction potential (−0.85 V vs NHE) than that of FNR (−0.54 V vs NHE), illustrating that the photoexcited electrons within Zr‐MOF can be efficiently shuttled to the FNR center for reduction of NAD^+^ into NADH. Furthermore, photoelectrochemical measurements were undertaken to scrutinize the electron transfer efficiency and separation dynamics. Fluorine‐doped tin oxide (FTO) was chosen as the operative electrode, upon which the sample was deposited via a drop‐casting method. As depicted in Figure 3f, upon increasing the loading quantity of FNR under light illumination (420 nm cutoff filter), the FNR@Zr‐MOF exhibited an enhanced photocurrent response compared to that of Zr‐MOF, demonstrating the most efficient electron/hole dissociation.

### 2.4 Photocatalytic NADH Regeneration

**Figure 4:**
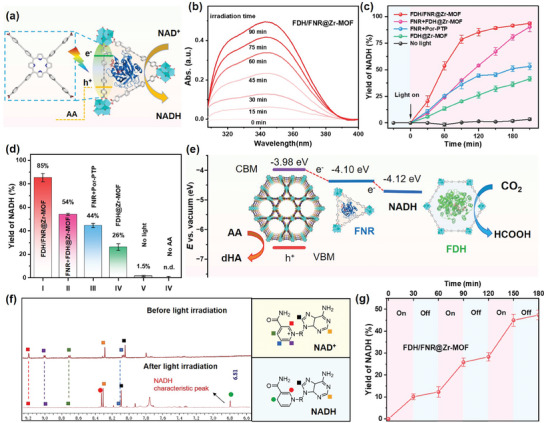
a) Graphic representation for photoexcited NADH generation over FDH/FNR@Zr‐MOF. b) Photoexcited NADH generation of FDH/FNR@Zr‐MOF system versus irradiation time (at 0, 15, 30, 45, 60, 75, and 90 min) from UV–vis spectra after ten times dilution. c) Yield of NADH versus irradiation time for FDH/FNR@Zr‐MOF, FNR+FDH@Zr‐MOF, FNR+Por‐PTP, FDH@Zr‐MOF and no light. d) The yield of NADH over different group after irradiation for 120 min. e) The energy diagram for the electron transfer cascade in the FDH/FNR@Zr‐MOF. f) ^1^H NMR measurement of NAD^+^ before (up) and after (down) light irradiation. g) Continuous NADH generation by cycling light “on” and “off.” 30 min for each period.

As is well‐known, FNR serves as the electron mediator within the photosynthetic electron transport chain, where it receives electrons from ferredoxin (Fd) and H^+^ in the matrix, subsequently reducing NAD(P)^+^ to NAD(P)H.^[^ ^40^ ^]^ Such photoelectric outcomes have promoted us to contemplate whether it possesses the ability to receive photoexcited electrons from photosensitive Zr‐MOF, thereby reducing NAD^+^ in enzymatic factories geared toward the production of high‐value‐added products. In this study, photocatalytic NADH regeneration within the FDH/FNR@Zr‐MOF system was initially executed in Tris buffers (0.5 m, pH 7.4) utilizing ascorbic acid (AA), Xenon lamp equipped with 420 nm filter and oxidative state NAD^+^ (1.0 mm) as the electron sacrificial agent, light sources, and the energy‐carrying cofactor, respectively (**Figure** **4**a). UV–vis spectra were employed to track the yield of photogenerated NADH leveraging its maximal absorbance at 340 nm. The concentration of NADH was calibrated using the Lambert‐Beer law according to its own molar extinction coefficient (*ε*) at 340 nm appropriately 6.22 × 10^3^ L mol^−1^ cm^−1^ (Figure S20, Supporting Information). The calibrated NADH standard curve with the curve equation of *A* = 6.21 × *C* (mM) + 7.6 × 10^−4^ (R > 0.999) can also be well matched with the results (Figures S21 and S22, Supporting Information).

Upon irradiation with the Xe light (420 nm cutoff filter, 93 mW cm^−2^), the absorbance at 340 nm gradually increased with the duration in irradiation time (at 0, 15, 30, 45, 60, 75, and 90 min) for the FDH/FNR@Zr‐MOF system (Figure 4b), indicating the successful photogeneration of NADH cofactor. The photocatalytic process was subsequently scrutinized through the meticulous monitoring of dynamic curves pertaining to various group sets. As depicted in Figure 4c, during the 4 h measurement procedure, the FDH/FNR@Zr‐MOF system exhibited a saturation kinetic profile, with the NADH yield progressively rising and ultimately attaining equilibrium. To quantitively analyze the catalytic efficiency, we extracted the NADH yield of different groups at time point 120 min and listed in Figure 4d. It was discovered that more than 85% of NAD^+^ underwent conversion to NADH in the presence of FDH/FNR@Zr‐MOF (I), markedly contrasting with the significantly lower percentages of 54% for ex‐encapsulated FNR group (FNR+FDH@Zr‐MOF, II) and 44% for non‐MOF system (FNR + POR‐PTP, III), respectively. We believe that the primary reason was MOF encapsulation can facilitate the electron transfer route from MOF frameworks to FNR.

In the case of the system devoid of FNR (FDH@Zr‐MOF, IV), the NADH yields attain a maximum of 26% when relying solely on direct electron transfer from the MOF to the NAD^+^ cofactor. To confirm the NADH generation is due to the photocatalysis process, two additional assays, in the absence of light (V) and in the absence of AA (IV) were conducted. Notably, NADH was barely detectable in the above systems. Meanwhile, the reduction potential of FNR (‐4.10 eV versus vacuum level) was situated between *E*_CB_ of Zr‐MOF and NADH,^[^ ^41^ ^]^ an energy diagram for the electron transfer cascade within the FDH/FNR@Zr‐MOF system was presented in Figure 4e. All these outcomes definitively corroborated our hypothesis that FNR as a pivotal electron mediator in our system can facilitate the thermodynamically favorable transfer of photogenerated electrons from Zr‐MOF frameworks to NAD^+^.

To monitor the chemical structure of final product, the reaction mixture was centrifuged and the supernatant was checked with ^1^H NMR. As illustrated in Figure 4f, upon Xe light irradiation, the characteristic peaks (9.2, 9.0, 8.7, and 8.1 ppm) associated with the pyridyl ring of NAD^+^ vanished completely, concomitant with the emergence of a novel peak at 6.8 ppm (indicated by the green dot) after the photocatalytic reaction. This newly observed peak is attributed to the unique signature of the reduced NADH cofactor. Meanwhile, the conversion efficiency of NADH in the photocatalysis system was associated with the concentration of electron sacrificial agent AA (Figure S23, Supporting Information), the amount of encapsulated FNR (Figure S24, Supporting Information) and the power density of Xe light (Figure S25, Supporting Information). In addition, by running light on/off cycles, as depicted in Figure 4g, FDH/FNR@Zr‐MOF catalytic system exhibited photoregulated NADH accumulation with repeated “on‐off” cycles. According to the reported method, the apparent quantum yield (AQY) over FDH/FNR@Zr‐MOF was calculated to be 1.2% under 420 nm monochromatic light illumination.^[^ ^12^ ^]^ All of these results instilled confidence in us that the FDH/FNR@Zr‐MOF hybrids could potentially be utilized for cofactor‐mediated CO_2_ fixation.

### 2.5 Photocatalytic CO_2_ Fixation

**Figure 5:**
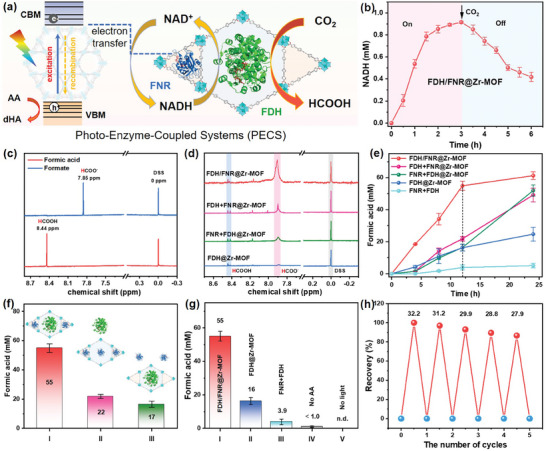
a) Graphic representation for photocatalytic CO_2_ fixation over FDH/FNR@Zr‐MOF. b) NADH generation over FDH/FNR@Zr‐MOF under light irradiation and then CO_2_ saturation without light. c) ^1^H NMR measurement of HCOOH and HCOO^−^. d) ^1^H NMR measurement of photocatalytic product over FDH/FNR@Zr‐MOF, FDH+FNR@Zr‐MOF, FNR+FDH@Zr‐MOF and FDH@Zr‐MOF. e) The concentration of product versus irradiation time for FDH/FNR@Zr‐MOF, FDH+FNR@Zr‐MOF, FNR+FDH@Zr‐MOF, FDH@Zr‐MOF and FDH+FNR. f) The concentration of product at 12 h for FDH/FNR@Zr‐MOF, FDH+FNR@Zr‐MOF and FNR+FDH@Zr‐MOF. g) The concentration of generated formic acid at 12 h for FDH/FNR@Zr‐MOF, FDH@Zr‐MOF, FNR+FDH, no AA and no light. h) Recyclability of FDH/FNR@Zr‐MOF for CO_2_ fixation in five cycles. During each cycle, the system underwent irradiation with Xe light for a duration of 8 h for the photocatalysis process.

The integration of FNR and *Cb*FDH within Zr‐MOF provided the feasibility for tandem cascade reactions for CO_2_ fixation. Upon integrating the photocatalysts (light reaction) with the biocatalysts (dark reaction), such a system can efficiently emulate the natural photosynthesis procedure of light absorption, electron transfer, and substrate conversion into valuable chemical products. As illustrated in **Figure** **5**a, the photogenerated NADH within the FDH/FNR@Zr‐MOF system was able to be directly utilized by *Cb*FDH for conversion of CO_2_ species into formic acid, with the subsequent oxidation of NADH into NAD^+^ to sustain cofactor recycling. To validate this, we bubbled CO_2_ into the FDH/FNR@Zr‐MOF system, which had been irradiated with Xe light for 3 h for NADH generation. As depicted in Figure 5b, it was observed the generated NADH underwent gradual consumption in the absence of light irradiation, indicating that NADH might undergo oxidation to NAD^+^ in the FDH/FNR@Zr‐MOF system, concurrently converting CO_2_ into to formic acid.

To quantitatively analyze the amount of photogenerated formic acid, ^1^H NMR measurement was employed with 4,4‐dimethyl‐4‐silapentane‐1‐sulfonic acid (DSS, δ = 0 ppm) as an external standard. In an aqueous medium, the produced formic acid may exist in the acid form (HCOOH) and alkaline form (HCOO^−^). ^1^H NMR results indicated the resonance peaks of **H**COOH and **H**COO^−^ were located at 7.9 and 8.4 ppm, respectively (Figure 5c,d). In the following description, we will directly use formic acid to describe the total generation of formic acid and formate species and the concentration was measured using the following equation:

(1) $$C=N\times C_{\mathrm{DSS}}\times \frac{A_{7.9}+A_{8.4}}{A_{\mathrm{DSS}}}$$

where *C* and *C*_DSS_ were concentrations of generated formic acid and external standard, respectively. *A*_7.9_, *A*_8.4_, and *A*_DSS_ are the integrated areas at 7.9, 8.4 and 0.0 ppm, respectively. N is the number of H atoms of the silylmethyl group over DSS (N = 9).

To detect the photoexcited formic acid generation across various group sets, the product concentration versus irradiation time profiles were carried out. As depicted in Figure 5e, the FDH/FNR@Zr‐MOF system exhibited the highest CO_2_ conversion efficiency during the reaction period. Due to the significant constraint imposed by the limited availability of CO_2_ (photocatalytic reaction occurring in an enclosed environment), the catalytic conversion efficiency was considerably hindered after 12 h, resulting in a modest increase in the quantity of formic acid produced. Herein, we collected the results at 12 h for comparison and the results were listed in Figure 5f. By optimizing the pathways of substrate transfer, it was disclosed that the generated formic acid (HCOOH + HCOO^−^) for the FDH/FNR@Zr‐MOF system attained an optimal concentration of appropriately 55 mm and a corresponding evolution rate of 4580 µmol g^−1^ h^−1^ within a span of 12 h reaction, surpassing the performance of most previously documented FDH immobilized systems (Table S1, Supporting Information). In contrast, this value could only reach to ≈22 mm for the FDH+FNR@Zr‐MOF system and 17 mm for the FNR+FDH@Zr‐MOF system, respectively, which is 2.5 and 3.4 times lower than that of the FDH/FNR@Zr‐MOF system, respectively.

Moreover, a comparative analysis was conducted through the execution of six distinct assays, FDH/FNR@Zr‐MOF (I), FDH@Zr‐MOF (II), FNR+FDH (III), no AA (IV) and no light (V), in order to delve into the underlying mechanism of CO_2_ conversion. As depicted in Figure 5g, the generation of photoelectrons from Zr‐MOF serves as a pivotal role in photo‐enzymatic catalysis for CO_2_ fixation in the FDH/FNR@Zr‐MOF system. After light irradiation, photoexcited electrons originating from Zr‐MOF structure were efficiently transferred to adjacent FNR enzymes for reduction of NAD^+^ cofactor into NADH, which could be subsequently captured by nearby *Cb*FDH enzyme for enabling CO_2_ fixation into HCOOH while NADH itself oxidized to NAD^+^ for recycling. The absence of each component, including FNR (II, 16 mm), Zr‐MOF (III, 3.9 mm), electron sacrificial agent (IV, < 1.0 mm), and light (V, n.d.), would notably hinder the efficiency of the photocatalytic conversion process. Hence, the confinement of tandem enzymes within MOF channels enhanced the localized NAD^+^/NADH and expedited the substrate transformation between FNR and *Cb*FDH, thus ultimately augmenting the overall CO_2_ conversion efficiency into formic acid. Notably, the FDH/FNR@Zr‐MOF system can be effortlessly isolated from the reaction systems via centrifugation to ensure its reusability. More than 86% CO_2_ conversion efficiency remained even after five consecutive cycles (Figure 5h), underscoring its robust performance. These accomplishments motivate researchers to deliver more efficacious PECSs toward producing high‐value products or intermediates in an environmentally benign manner.

## 3 Conclusion

In summary, we developed a mesoporous Zr‐MOF from Por‐PTP and Zr_6_ cluster which possesses hierarchical channels for co‐encapsulation of both *Cb*FDH and FNR for cofactor‐dependent photoenzymatic CO_2_ conversion. A spatiotemporal encapsulation strategy was devised to enhance the encapsulation efficiency of both enzymes while ensuring a precise distribution throughout the materials. The large hexagonal channel and moderate triangular channel are uniquely designed to sequentially accommodate *Cb*FDH and FNR, respectively, in a specific order (*Cb*FDH first, then FNR), which was designated as FDH/FNR@Zr‐MOF. Upon illumination, photogenerated electrons were efficiently channeled to the encapsulated FNR center within MOF pores for the reduction of NAD^+^ into NADH, whereas the accompanying photogenerated holes were consumed by sacrificial agent AA for ensuring electric neutrality. Meanwhile, the locally generated NADH traversed the orthogonal windows to navigate toward the nearby *Cb*FDH for the enhanced conversion of CO_2_ into formic acid. The strategic encapsulation of tandem enzymes within MOF significantly amplified the local NAD^+^/NADH and streamlined the substance transformation process in FDH/FNR@Zr‐MOF system, leading to a notable enhancement in the ultimate CO_2_ conversion efficiency (55 mm at 12 h). Crucially, the FDH/FNR@Zr‐MOF demonstrated remarkable catalytic efficiency, preserved stability and excellent recyclability. By merging the principles of artificial photosynthesis, multienzyme cascade reactor, and green manufacturing, this research presents a paradigm for establishing a novel photo‐enzyme factory that caters to the high demand for the production of valuable chemicals and pharmaceuticals.

## 4 Experimental Section

### Synthesis and Characterization of Por‐PTP Ligand

The synthesis procedure of MOF ligand, *meso*‐tetrakis‐(4‐((phenyl)ethynyl)benzoate)porphyrin (Por‐PTP), was modified from the previously reported literature,^[^ ^36^ ^]^ and the detailed synthesis process was shown in Supporting Informations.

### Preparation of Channel‐type Zr‐MOF

Zirconium oxychloride octahydrate (ZrOCl_2_·8H_2_O, 200 mg, 0.62 mmol) and trifluoroacetic acid (TFA, 743 µL, 10 mmol) were mixed in DMF (50 mL) and sonicated for dissolve. The clear solution was then incubated in an oven at 80 °C for 1 h. the solution was cooled down within 30 min to give the storage solution A. Then the Por‐PTP (35.76 mg, 0.03 mmol) dissolved in dry DMF (50 mL) was added into the storage solution A to get a dark green solution. After sonication for 10 min, the mixture solution was heated in an oven at 80 °C for 3 h to give a dark‐purple solid power. The solid was separated from the liquid and washed with fresh DMF for three times to give the Zr‐MOF.

### Expression and Purification of CbFDH and FNR

Briefly, the gene sequences of *Cb*FDH or FNR were amplified by PCR and cloned into pET28a(+) vector to give their recombinant plasmids. After confirmation, it was transformed into *E. coli* BL21(DE3) ultracompetent cell to give recombinant bacteria, and grew in 1 L luria‐bertani (LB) medium with shaking at 37 °C. After OD_600_ was reached to 0.8, protein expression was induced with the addition of isopropyl‐beta‐D‐thiogalactopyranoside (IPTG, 1.0 mm). After ultrasonication and centrifugation, the supernatant was purified by Ni‐NTA His‐tag purification agarose. Purified *Cb*FDH or FNR power was harvested after dialysis and lyophilization, which was confirmed by SDS‐PAGE and enzymatic catalytic assay.

### The Spatiotemporal Encapsulation of Tandem Enzymes within Zr‐MOF

FDH/FNR@Zr‐MOF was defined as the stepwise encapsulation of tandem enzymes with an order of *Cb*FDH and FNR. Generally, 1.0 mg of activated Zr‐MOF was added into *Cb*FDH solution (4.5 mg mL^−1^), which was further incubated at 4 °C for 12 h. The nanoparticle was centrifuged and washed with ultrapure water three times. Then, FNR solution (4.5 mg mL^−1^) was added and incubated at 4 °C for another 12 h. The nanoparticle was centrifuged and washed with ultrapure water for three times to get FDH/FNR@Zr‐MOF. For FNR&FDH@Zr‐MOF, the stepwise encapsulation processes took place with order of FNR and *Cb*FDH.

### Photocatalytic Regeneration of NADH

The photocatalytic regeneration of NADH from NAD^+^ was carried out in a quartz reactor at room temperature. The photocatalytic regeneration of NADH was conducted in 5 mL Tris‐buffer (0.5 m, pH 7.4) containing NAD^+^ (1.0 mm) as a cofactor, ascorbic acid (AA, 200 mm) as a sacrificial agent, and different photocatalysts (5.0 mg). A 300 W xenon lamp equipped with a 420 nm cutoff filter was used as a light source (CEL‐PF300‐T8, CEAULIGHT, Beijing, China). The reaction system was first incubated under dark conditions for 30 min to balance adsorption and desorption and then was exposed to light. The photogenerated NADH was quantitatively calculated through the NADH standard curve at 340 nm.

### The Artificial Photosynthesis of Formic Acid From CO_2_

The photosynthesis of formic acid from CO_2_ was performed within the quartz cuvette reactor at room temperature, using a 300 W xenon lamp with a 420 nm cutoff filter as a light source. Generally, 5 mL tris‐buffer (0.5 m, pH 7.4) containing NAD^+^ (1.0 mm) as cofactor, ascorbic acid (AA, 200 mm) as a sacrificial agent, and different photocatalysts (5.0 mg) was used as reaction mixture and was bubbled with CO_2_ gas for 0.5 h before use. A 300 W xenon lamp (CEL‐PF300‐T8, CEAULIGHT, Beijing, China) equipped with a 420 nm cutoff filter was used as a light source. The photogenerated formate spesis was quantitatively calculated using ^1^H NMR measurement with DSS as the internal standard.

### Quantitative Calculation of Photogenerated Formic Acid

At time intervals, 400 µL of sample was taken out and mixed with 50 µL of DSS (5.0 mm) and 50 µL of D_2_O for measuring formic acid using ^1^H NMR measurement. The final concentration of formic acid was determined using the following equation:

(2) $$C=\frac{5}{4}\times N_{H,\;\;\mathrm{DSS}}\cdot C_{\mathrm{DSS}}\cdot \frac{A_{\delta =7.9}+A_{\delta =8.4}}{A_{\delta =0}}$$

In which ***C*** is the concentration of photogenerated formic acid which exists in the form of formic acid and formate in NMR solution. ***N***_H, DSS_ NDS and ***C***_DSS_ are the number of H in DSS molecule (N = 9) and the concentration of DSS in solution (0.5 mm), respectively. ***A***_δ=0_, ***A***_δ=7.9_, and ***A***_δ=8.4_ are the integral area at δ = 0 for DSS, δ = 7.9 for formate, and δ = 8.4 for formic acid, respectively.

## Conflict of Interest

The authors declare no conflict of interest.

## Supporting information



Supporting Information

## Data Availability

The data that support the findings of this study are available from the corresponding author upon reasonable request.
