# Supplementary material for: Spatiotemporal Encapsulation of Tandem Enzymes in Hierarchical Metal–Organic Frameworks for Cofactor‐Dependent Photoenzymatic CO2 Conversion
Source: Adv Sci (Weinh). 2024 Nov 8;11(48):2410024. doi: 10.1002/advs.202410024 (PMC11672261; doi:10.1002/advs.202410024)
Supplement: Supplementary file 1 — Supporting Information [file ADVS-11-2410024-s001.docx]

((Supporting Information can be included here using this template))

Supporting Information

Spatiotemporal encapsulation of tandem enzymes in hierarchical metal-organic frameworks for cofactor-dependent photoenzymatic CO_2_ conversion

Yan Li, Jieqiong Wang, Xiaoqian Shi, Xiaoxuan Yu, Shuangjiang Yu, Junqiu Liu*, and Hongcheng Sun*

Key Laboratory of Organosilicon Chemistry and Material Technology, Ministry of Education, College of Material, Chemistry and Chemical Engineering, Hangzhou Normal University
Hangzhou, 311121, China

E-mail: junqiuliu@jlu.edu.cn (J. Liu); sunhc@hznu.edu.cn (H. Sun)

Keywords:

Spatiotemporal encapsulation; tandem enzymes; metal-organic frameworks (MOFs); photoenzymatic catalysis; CO_2_ fixation.

**Content**

Experimental Section 3

1. Chemicals and Materials 3

2. Characterizations 3

3. Synthesis of ligand *meso*-tetrakis-(4-((phenyl)ethynyl)benzoic acid)porphyrin (Por-PTP) 4

*3.1 Synthesis of tert-butyl 4-iodobenzoate (tBut-IBz, 1)* 4

*3.2 Synthesis of tert-butyl 4-((4-formylphenyl)ethynyl)benzoate (tBut-BzH, 2)*: 5

*3.3 Synthesis of meso-tetrakis-(tert-butyl 4-((phenyl)ethynyl)benzoate)porphyrin (tBut-Por-PTP, 3)* 7

*3.4 Synthesis of meso-tetrakis-(4-((phenyl)ethynyl)benzoate)porphyrin (Por-PTP, 4)* 9

4. Preparation of Zr-MOF 10

5. Expression and purification of Enzymes 11

*5.1 Formate dehydrogenase from Candida boidinii (CbFDH):* 11

*5.2 Ferredoxin-NADP+ reductase from Synechococcus sp. (FNR):* 12

6. Spatiotemporal encapsulation of FNR and *Cb*FDH within Zr-MOF 14

*6.1 FDH/FNR@Zr-MOF* 14

*6.2 FNR/FDH@Zr-MOF* 14

Measurements 14

1. The CbFDH activity assay 14

2. Stability of FDH@Zr-MOF in harsh conditions 15

3. The degradation of Zr-MOF and release of enzymes 15

4. Thermogravimetric analyses (TGA) measurements 15

5. Confocal laser scanning microscopy (CLSM) measurements 15

6. Solid UV-visible diffuse reflection spectroscopy (UV-vis DRS) 15

7. Electrochemical and photocurrent measurements 16

8. Photocatalytic regeneration of NADH 16

9. NADH production *via* alternatively light on/off cycles 17

10. The artificial photosynthesis of formic acid from CO_2_ 17

11. The Apprent Quantum Yield Measurement (AQY) 17

Results and Characterization 19

References 27

**Experimental Section**

**1. Chemicals and Materials**

4-Iodobenzoic acid (I-BenA, 98%), 4-ethynylbenzaldehyde (Ey-BzH, 98%), potassium tert-butoxide (KO*t*But, 98%), copper(l) iodide (CuI, 99.99%), bis(triphenylphosphine)palladium dichloride (Pd(PPh_3_)_2_Cl_2_, 98%), 1,1'-bis(diphenylphosphino)ferrocene]dichloropalladium(II) (Pd(dppf)Cl_2_) were purchased from Aladdin Chemical Reagent Co. Ltd (Shanghai, China). Zirconium oxychloride octahydrate (ZrOCl_2_·8H_2_O), thionyl chloride (SOCl_2_, 99%), benzoic acid (BenA, 99%), trifluoroacetic acid (TFA, 99%) and pyrrole (99%)were bought from Energy Chemical. Reduced nicotinamide adenine dinucleotide (NADH), oxidized nicotinamide adenine dinucleotide (NAD^+^), ascorbic acid (AA) were purchased from Sigma-Aldrich (USA). Various kinds of solvent such as n-hexane, tetrahydrofuran (THF), dichloromethane (CH_2_Cl_2_), chloroform (CHCl_3_), N,N-Dimethylformamide (DMF), propionic acid were purchased from Sinopharm. Group Co., LTD and dried before use. (Shanghai, China). Fluoresceine isothiocyanate (FITC), coumarin-6-sulfonyl chloride (CSC, 97%) were purchased from Bide Pharmatech. Ltd. Isopropyl-beta-D-thiogalactopyranoside (IPTG), yeast extract, tryptone were purchased from Sangon Biotech (Shanghai) Co., Ltd. Pyrrole was distilled prior to use.

**2. Characterizations**

The molecular structures of synthesized organic linkers were identified by ^1^H NMR and ^13^C NMR using Bruker DMX500 spectrometer. The molecular weight was analyzed using tandem quadrupole (triple quadrupole) mass spectrometry (Agilent 1290-microTOF-Q Ⅱ) and matrix-assisted laser desorption ionization time-of-flight mass spectrometry (MALDI-TOF, Microflex). The hydrodynamic diameter (*D*_h_), size distribution and the zeta-potential of the materials were measured by dynamic light scattering (DLS) measurements using Zetasizer Nano ZSE. Fourier transform infrared spectra (FT-IR) were recorded on VERTEX 70. The morphological structures of the MOFs were characterized by transmission electron microscope (TEM, HITACHI, HT7700) with 100 kV accelerating voltage and scanning electron microscope (SEM, HITACHI, S-4800). Magnified structures and EDX elemental mappings were measured using high resolution field emission transmission electron microscope (HR-TEM, FEI Tecnai G2 F20) with 200 kV accelerating voltage. UV-Vis diffuse reflection spectroscopy (UV-Vis-DRS) and UV-vis absorption spectroscopy (UV-Vis) were recorded on a Shimadzu UV-2600 spectrometer. Fluorescent spectroscopy and steady-state fluorescence spectroscopy were carried out by using Edinburgh FLS1000 fluorescence spectrometer. The cyclic voltammograms (CV) and photoelectrochemical characterization were recorded on an electrochemical workstation (Chenhua, CHI 760E). Confocal laser scanning microscopy (CLSM, LSM 900 equipped with Airyscan 2) was used to colocalization staining analysis for MOF hybrids. The concentration of formic acid was estimated by ^1^H NMR (Bruker DMX500) with 4,4-dimethyl-4-silapentane-1-sulfonic acid (DSS, δ = 0 ppm) as internal standard. Photocatalytic experiments were carried out by using CEL-HXF300 Xenon lamp equiped with UV-cut filter as light source.

**3.** **Synthesis of ligand *meso*-tetrakis-(4-((phenyl)ethynyl)benzoic acid)porphyrin (Por-PTP)**

The synthesis rout of the ligand **Por-PTP (4)** was shown in Scheme S1.

**Scheme S1.** Synthesis route of organic ligand **Por-PTP, 4**.

*3.1 Synthesis of tert-butyl 4-iodobenzoate (****tBut-IBz, 1****)*

The tBut-IBz (1) was synthesized using modified literature process.^1^ Briefly, to a 100 mL one-necked round bottom flask, 4-iodobenzoic acid (I-BzA, 4.96 g, 20 mmol) was dissolved in dry CH_2_Cl_2_ (15 mL). Excess amount of thionyl chloride (SOCl_2_, 14.5 mL, 200 mmol) and a few drops of DMF (20 μL) were added into the solution in ice-water bath. After that, the mixture was heated to reflux (around 75 °C) for 3 h under N_2_ atmosphere to get a clear yellow solution. After evaporating all of solvent and SOCl_2_, the resulting yellow solid power was further dissolved in dry CH_2_Cl_2_ (40 mL), and potassium tert-butoxide (KO*t*But, 2.289 g, 20.4 mmol) was slowly added under stirring at 0 ^o^C. The mixture was reacted at 50 °C for 12 h. after cooling down to room temperature, the solution was extracted three times with 100 mL CH_2_Cl_2_ (300 mL total). The organic layer was further dried with MgSO_4_, concentrated, and separated with 200-300 mush column chromatography (hexane/CH_2_Cl_2_=1:1) to get compound 1 (*t*But-IBz, 5.42 g, 89.2%). ^1^H NMR (500 MHz, CDCl_3_, Figure S1) δ = 7.76 (d, J = 8.4 Hz, 1H), 7.68 (d, J = 8.3 Hz, 1H), 1.58 (s, 9H). MS (Q-TOF, m/z, Figure S2): Calc. for C_11_H_13_IO_2_, 304.4; found: 327.0 ([M+Na]^+^).


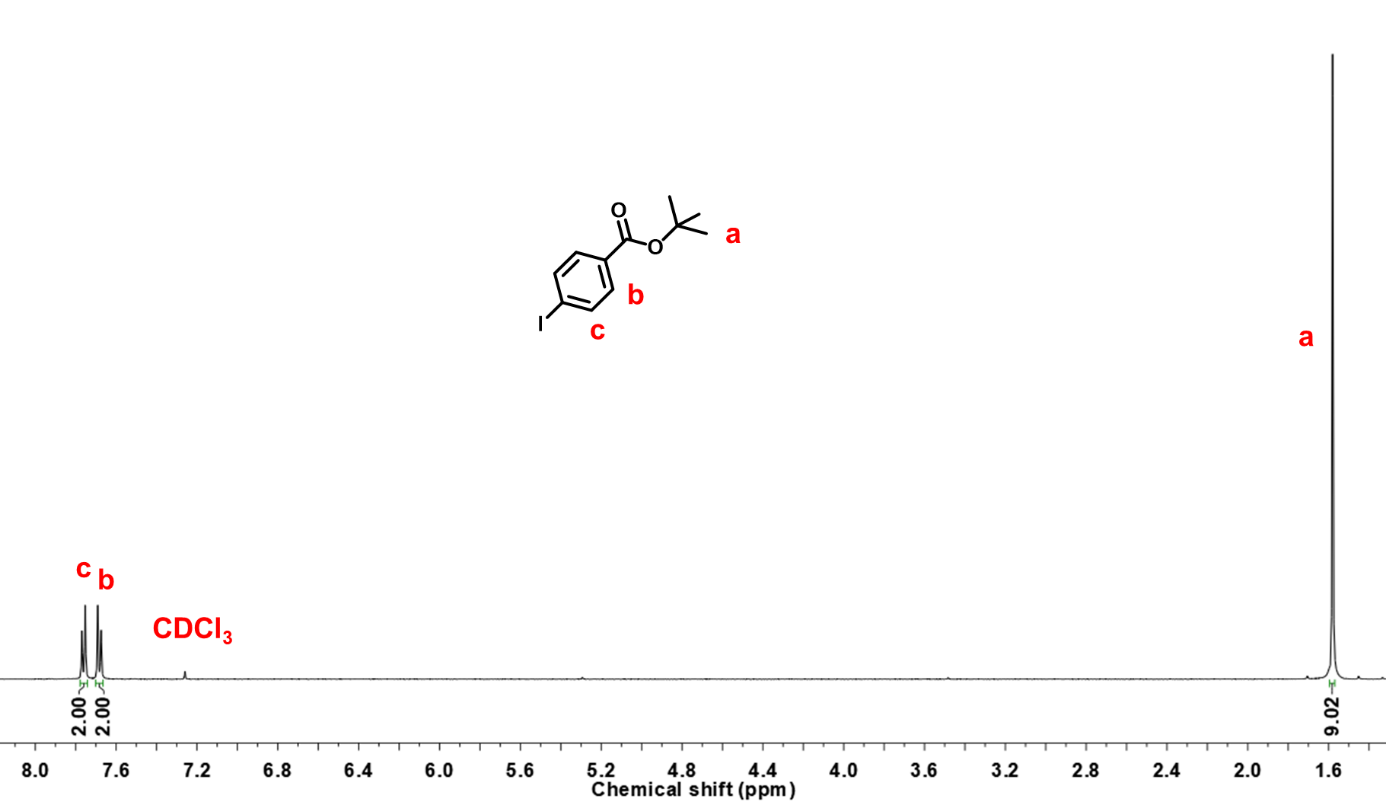


**Figure S1**. ^1^H NMR spectrum of compound 1, *t*But-IBz.


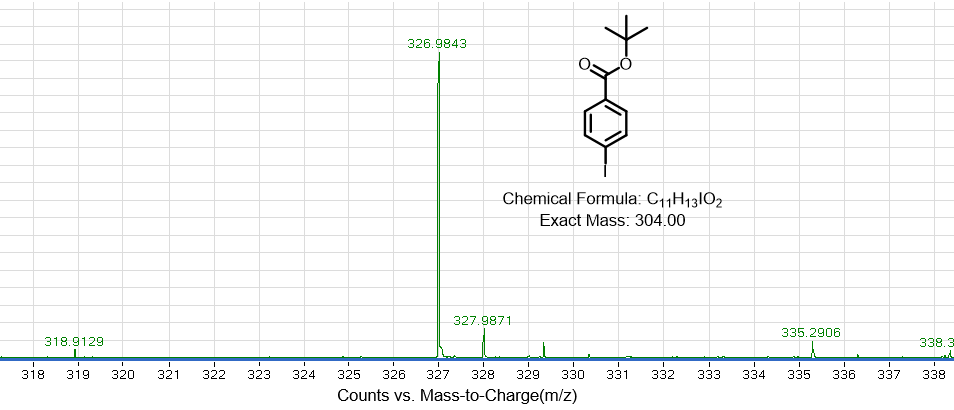


**Figure S2**. Mass spectrum of compound 1, *t*But-IBz.

*3.2 Synthesis of tert-butyl 4-((4-formylphenyl)ethynyl)benzoate (****tBut-BzH, 2****)*:

Compound 1 (*t*But-IBz, 912 mg, 3 mmol), 4-ethynylbenzaldehyde (Ey-BzH, 410.6 mg, 3.06 mmol,1.02 equiv), Pd (dppf) Cl_2_ (53.7 mg, 0.075 mmol, 2.5 mol%) and CuI (28.6 mg, 0.15 mmol, 5 mol %) were added into a 50 mL one-necked round bottom flask. Dry THF (15 mL) and dry TEA (7.5 mL) were added and then the mixture was degassed for 20 min. The solution was stirred at 45 ^o^C for 24 h under N_2_ atmosphere to give a dark black mixture. After filtration, and washed with large amount of THF (200 mL), the filter liquor was collected and dried by rotary evaporation. The solid was further purified with 200-300 mush column chromatography (hexane/CH_2_Cl_2_=1:1) to get white compound 2 (*t*But-BzH, 689 mg, 75.1%). ^1^H NMR (500 MHz, CDCl_3_, Figure S3) δ = 10.03 (s, 1H), 7.99 (d, 2H), 7.88 (d, 2H), 7.70 (d, 2H), 7.59 (d, 2H), 1.61 (s, 9H). MS (Q-TOF, m/z, Figure S4): Calc. for C_20_H_8_O_3_, 306.1; found: 329.1 ([M+Na]^+^).


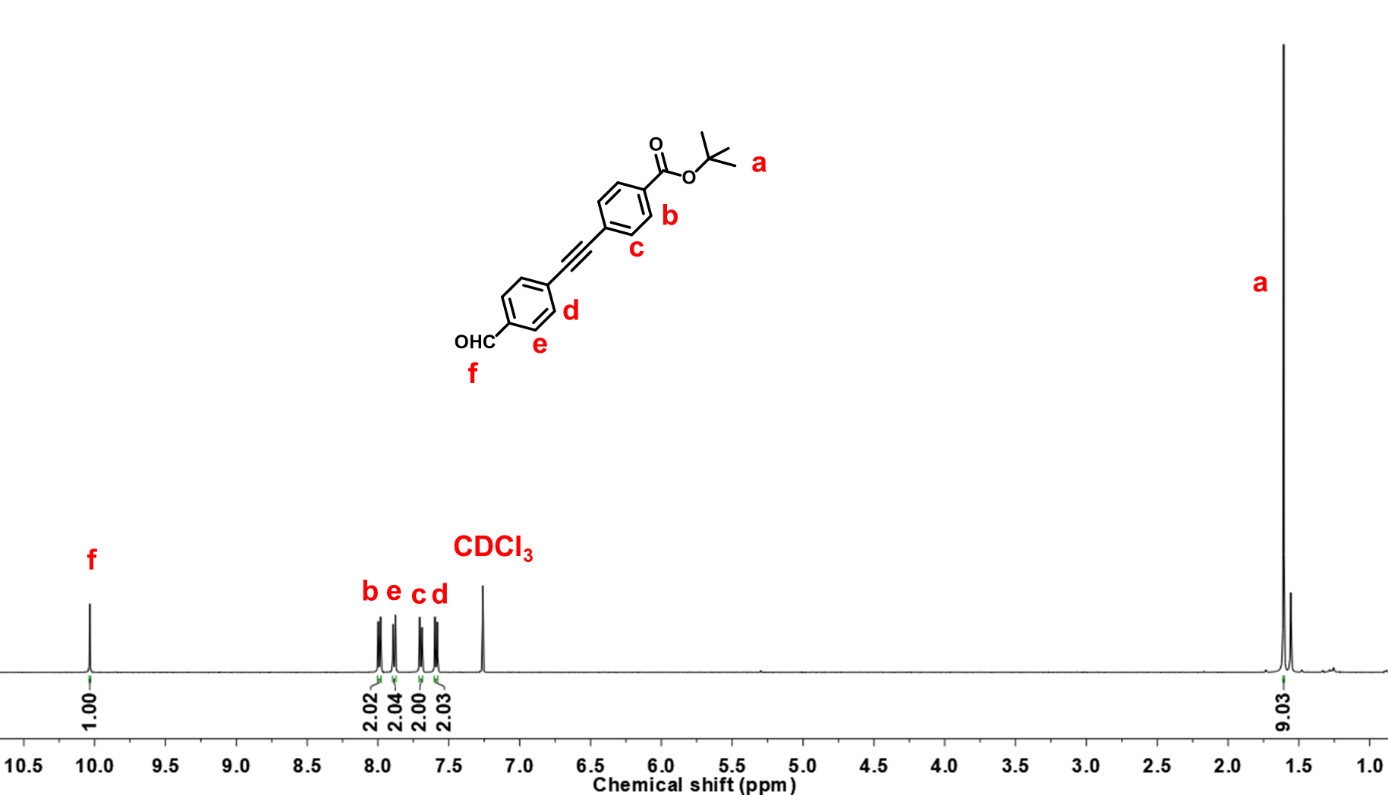


**Figure S3**. ^1^H NMR spectrum of compound 2, *t*But-BzH.


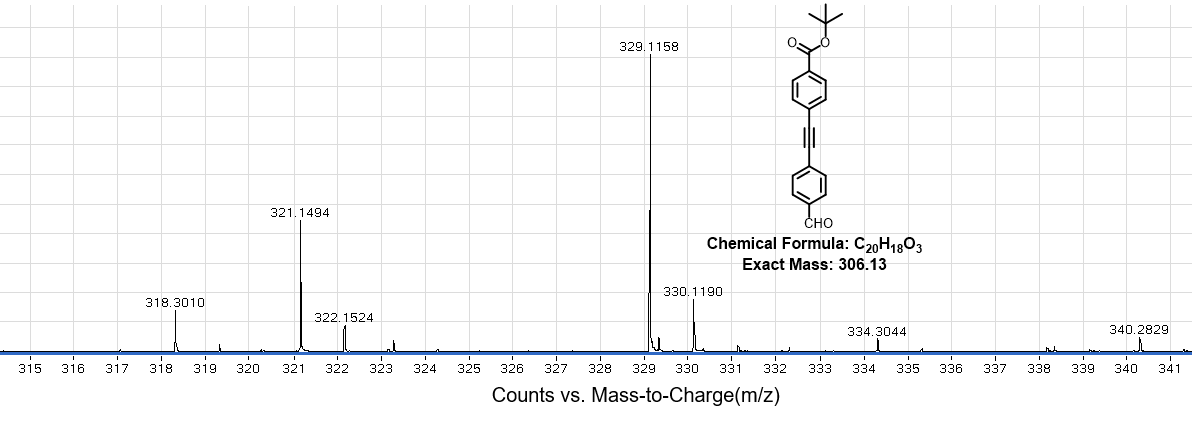


**Figure S4**. Mass spectrum of compound 2, *t*But-BzH.

*3.3 Synthesis of meso-tetrakis-(tert-butyl 4-((phenyl)ethynyl)benzoate)porphyrin (****tBut-Por-PTP, 3****)*

Compound 2 (*t*But-BzH, 1.226 g, 4 mmol) was dissolved in propionic acid (40 mL) and heated to reflux (140 ^o^C) under N_2_ atmosphere. The freshly distilled pyrrole (278 μL, 4 mmol, 1 equiv) was slowly dropcast into the reaction mixture within 30 min to give a black solution with red fluorescence. The mixture was further allowed to reflux for 3 h. After that, methanol (40 mL) was added inside the mixture after cooling and the crude mixture were stay up overnight at 4 ^o^C. After filtration, the filter plate was washed with methanol and water to give a crude purple solid. It was further purified with 200-300 mush column chromatography (CH_2_Cl_2_) to get purified purple compound 3 (*t*But-Por-PTH, 242 mg, 17.1%). ^1^H NMR (500 MHz, CDCl_3_, Figure S5) δ = 8.82 (s, 8H), 8.16 (d, J = 8.1 Hz, 8H), 7.99 (d, J = 8.4 Hz, 8H), 7.89 (d, J = 8.1 Hz, 8H), 7.65 (d, J = 8.4 Hz, 8H), 1.57 (s, 36H), -2.84 (s, 2H). MS (MALDI-TOF, m/z, Figure S6): Calc. for C_96_H_78_N_4_O_8_, 1414.6; found: 1415.7 ([M+H]^+^).


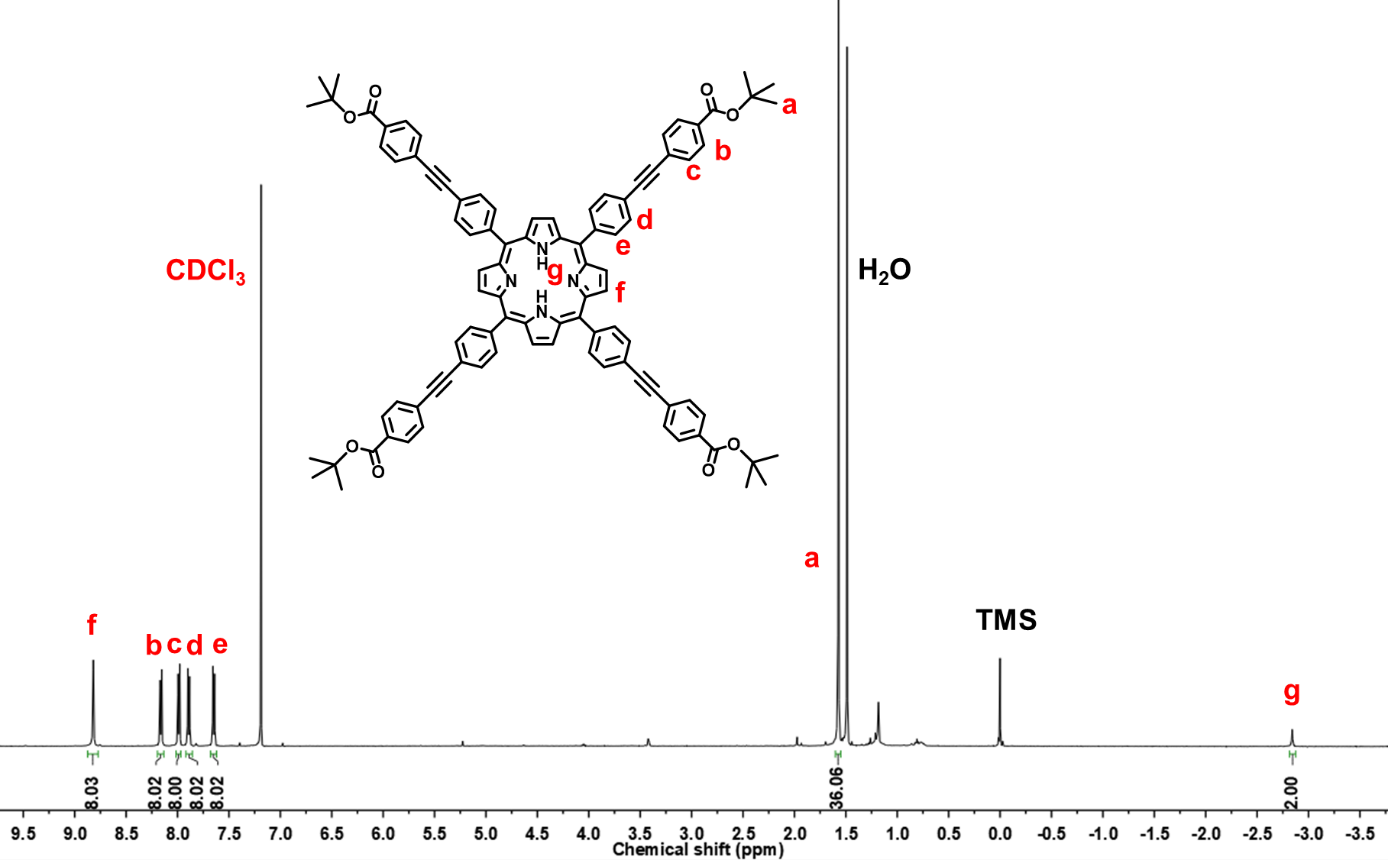


**Figure S5**. ^1^H NMR spectrum of compound 3, *t*But-Por-PTP.


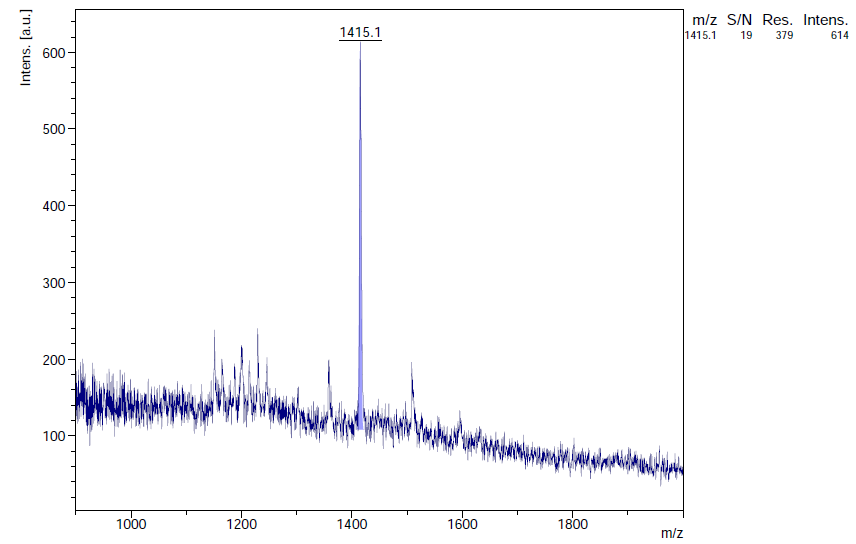


**Figure S6**. MALDI-TOF spectrum of compound 3, *t*But-Por-PTP.

*3.4 Synthesis of* *meso-tetrakis-(4-((phenyl)ethynyl)benzoate)porphyrin (****Por-PTP, 4****)*

Compound 3 (tBut-Por-PTP, 566.3 mg, 0.4 mmol) was dissolved in CH_2_Cl_2_ (20 mL) in 100 mL one-necked round bottom flask. Trifluoroacetic acid (TFA, 8 mL) in CH_2_Cl_2_ (20 mL) solution was slowly added into the solution with color changed from purple to green. After further reacted at 80 ^o^C at under N_2_ atmosphere for 24 h, the mixture was dried by rotary evaporation to get dark green compound 4 (Por-PTP, 448 mg, 94%). ^1^H NMR (500 MHz, DMSO-d6, Figure S7) δ = 13.23 (s, 4H), 8.90 (s, 8H), 8.28 (s, 1H), 8.05 (t, J = 8.8 Hz, 16H), 7.82 (d, J = 7.7 Hz, 8H), -2.92 (s, 2H). MS (MALDI-TOF, m/z, Figure S8): Calc. for C_80_H_46_N_4_O_8_, 1190.3; found: 1189.5 ([M-H]^-^).


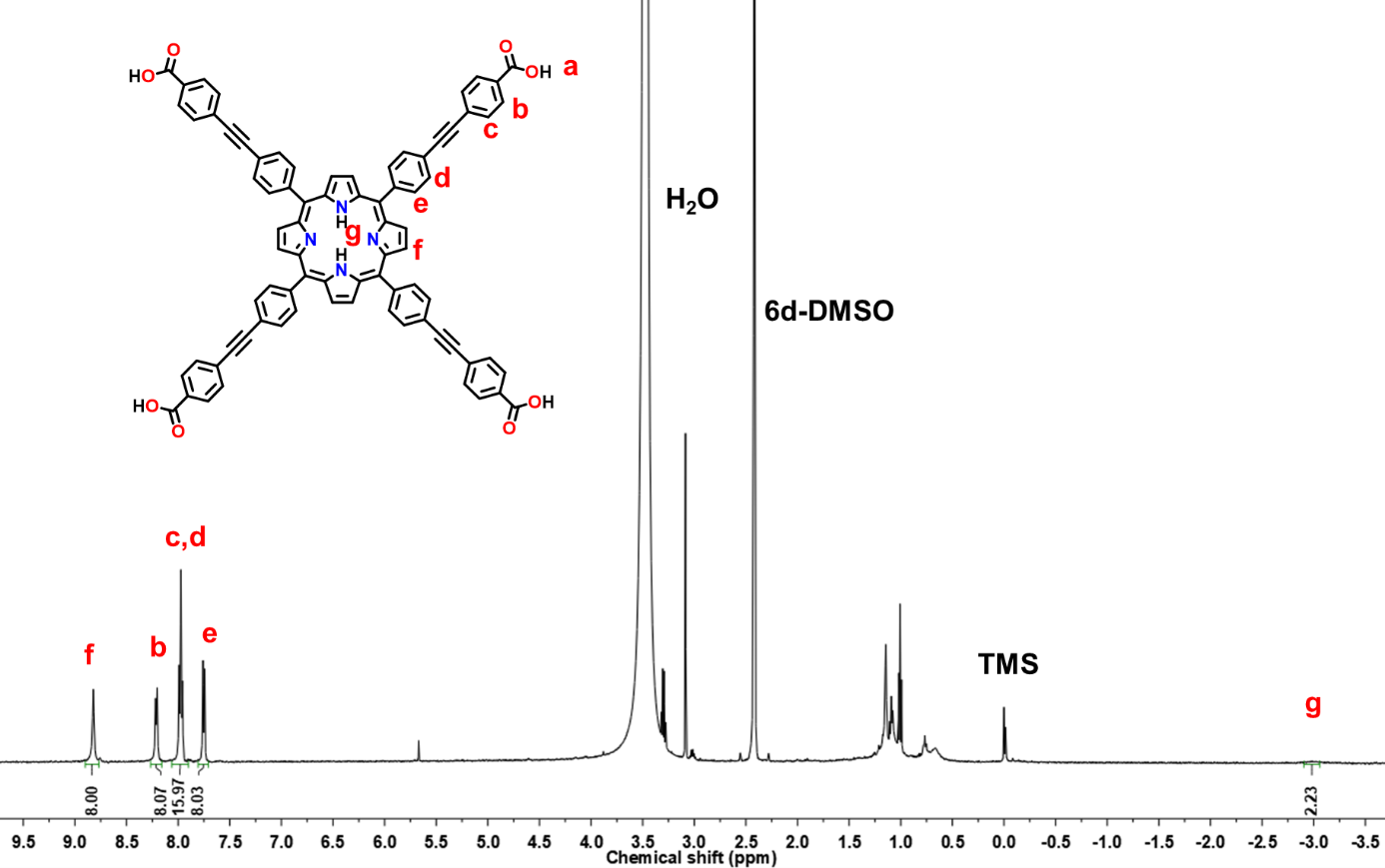


**Figure S7**. ^1^H NMR spectrum of compound 4, Por-PTP.


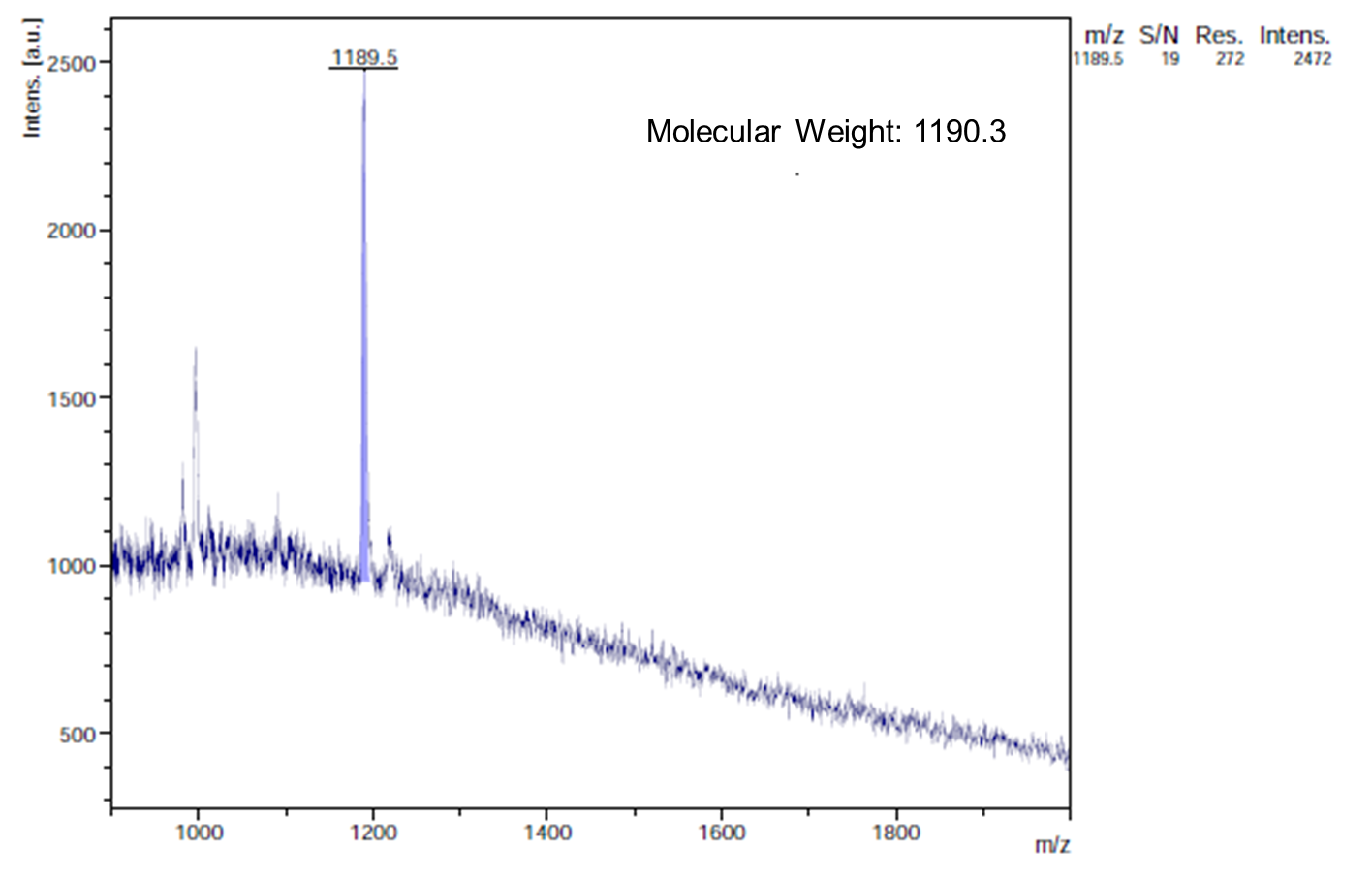


**Figure S8**. MALDI-TOF spectrum of compound 4, Por-PTP.

**4. Preparation of Zr-MOF**

Zirconium oxychloride octahydrate (ZrOCl_2_·8H_2_O, 200 mg, 0.62 mmol) and trifluoroacetic acid (TFA, 743 μL, 10 mmol) were mixed in DMF (50 mL) and sonicated for dissolve. The clear solution was then incubated in an oven at 80 ˚C for 1h. the solution was cooled down within 30 min to give the storage solution A.

The Por-PTP (35.76 mg, 0.03 mmol) dissolved in dry DMF (50 mL) was added into the storage solution A to get a dark green solution. After sonication for 10 min, the mixture solution was heated in an oven at 80 ^o^C for 3 h to give a dark-purple solid power. The solid was separated from liquid and washed with fresh DMF for 3 times to give the Zr-MOF. FT-IR was used to identified the chemical structures (Figure S9).


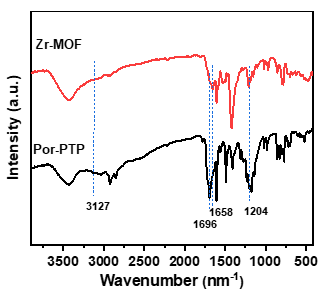


**Figure S9**. FT-IR of Por-PTP ligand and the synthesized Zr-MOF.

**5. Expression and purification of Enzymes**

*5.1 Formate dehydrogenase from Candida boidinii (CbFDH):*

Briefly, the gene sequence of formate dehydrogenase from *Candida boidinii* (EC.1.2.1.2; *Cb*FDH) through *in vitro* total gene synthesis was amplified by PCR and cloned into pET28a(+) vector to give a recombinant *Cb*FDH-pET28a(+) plasmid (Figure S10), which was further confirmed by DNA sequencing (Sangon). After confirmation, it was transformed into *E. coli* BL21(DE3) to give a recombinant bacteria (*E. coli* BL21(DE3)/*Cb*FDH-pET28a(+)), and grew in 1 L luria-bertani medium with shaking at 37 ^o^C. After OD600 was reached to 0.8, protein expression was induced at 30 ^o^C for 4 h with the addition of isopropyl-beta-D-thiogalactopyranoside (IPTG, 1.0 mM). After ultrasonication and centrifugation, the supernatant was purified by Ni-NTA His-tag purification agarose. A purified NAD^+^-dependent *Cb*FDH power was get after dialysis and lyophilization, which was confirmed by SDS-PAGE (Figure S12) and enzymatic catalytic assay.

The *Cb*FDH protein sequence was shown as follow:

MKIVLVLYGAGKHAADEEKLYGCTENKLGIANWLKDQGHELITTSDKEGGNSVLDQHIPDADIIITTPFHPAYITKERIDKAKKLKLVVVAGVGSDHIDLDYINQTGRKISVLEVTGSNVVSVAEHVVMTMLVLVRNFVPAHEQNINHDWEVAAIAKDAYDIEGKTIATIGAGRIGYRVLERLVPFNPKELLYYDYQALPKDAEEKVGARRVENIEELVAQADIVTVNAPLHAGTKGLINKELLSKFKKGAWLVNTARGAICVAEDVAAALESGQLRGYGGDVWFPQPAPKDHPWRDMRNKYGAGNATTPHYSGTTLDAQTRYAQGTKNILESFFTGKFDYRPQDIILLNGEYVTKAYGKHDKK


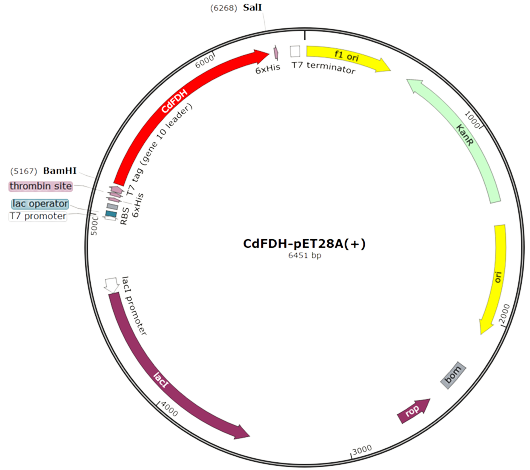


**Figure S10**. Plasmid structure of *Cb*FDH-Pet28a(+).

*5.2 Ferredoxin-NADP+ reductase from Synechococcus sp. (FNR):*

The gene sequence of ferredoxin-NADP+ reductase from *Synechococcus sp. (FNR)* through *in vitro* total gene synthesis was amplified by PCR and cloned into pET28(+) vector to get a recombinant FNR-pET28a(+) plasmid (Figure S11), which was further transformed into *E. coli* BL21(DE3) to a recombinant *E. coli* BL21(DE3)/FNR-pET28a(+) bacteria. It was grown up in 1 L luria-bertani medium with shaking at 37 ^o^C. After OD600 reached to 0.8, protein expression was induced at 28 ^o^C for 6 h with the addition of IPTG (1.0 mM). After ultrasonication and centrifugation, the supernatant was purified by Ni-NTA His-tag purification agarose and then was freeze-dried to give purified FNR protein. A purified NAD^+^-dependent *Cb*FDH power confirmed by SDS-PAGE (Figure S12).

The FNR protein sequence was shown as follow:

MVASAQKREVVVNLYRPNAPLIGQCVETYSLVGEGAPGLTKHIVLSLPDPNYRYLEGQSVGIIPPGVDDKGKPHKPRLYSIASTRYGDDGEGRTVSLSVKRAEYVDKETGQPGVGVCSGFLTDLKPGDEVMITGPSGKTFLLPEDENANLILIATGTGIAPFRAFIKHLFEEDPNYQGKIWLFFGVPTTSTLLYHGDLEAWKAQYGDRFRVDYAISREQQTPDGKKMYVQNRMAEYGPELWEMLQQPNTYTYICGLKGMEDGINSVMAPLAEQAGQDWSKFQKELKKANRWHEETY


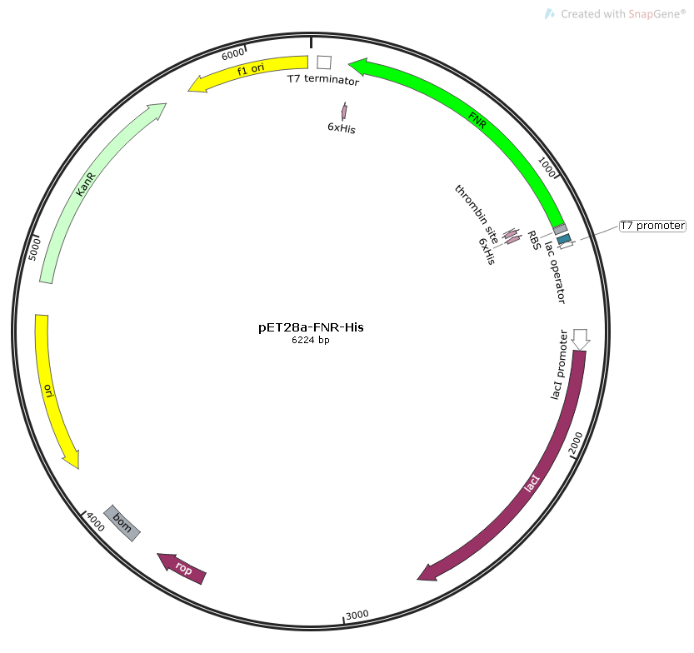


**Figure S11**. Plasmid structure of FNR-Pet28a(+).


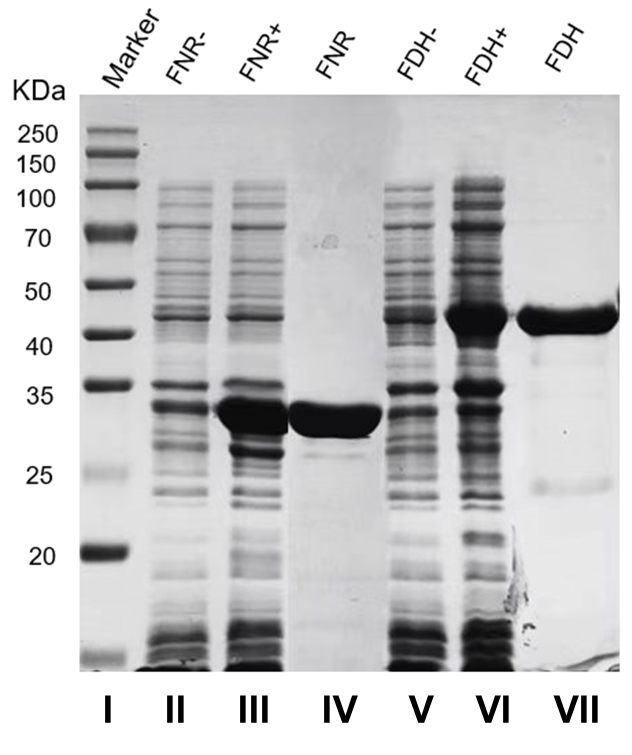


**Figure S12**. SDS-PAGE of *Cb*FDH and FNR expression and purification. Lane I: marker, Lane II: FNR before IPTG induction; Lane III: FNR after IPTG induction; Lane IV: FNR after Ni-NTA purification; Lane V: *Cb*FDH before IPTG induction; Lane VI: *Cb*FDH after IPTG induction; Lane VII: *Cb*FDH after Ni-NTA purification.

**6.** **Spatiotemporal encapsulation of FNR and *Cb*FDH within Zr-MOF**

*6.1 FDH/FNR@Zr-MOF*

FDH/FNR@Zr-MOF was defined as the spatiotemporal encapsulation of tandem enzymes with order of *Cb*FDH first and then FNR. Generally, 1 mg of activated Zr-MOF was added into *Cb*FDH solution (1.0 mL), which was furhter incubated at 4 ^o^C for 12 h. The nanoparticle was centrifuged and washed with ultrapure water for three times to get FDH@Zr-MOF. Then, FNR solution (1.0 mL) was added and incubated at 4 ^o^C for another 12 h. The nanoparticle was centrifuged and washed with ultrapure water for three times to get FDH/FNR@Zr-MOF.

*6.2 FNR/FDH@Zr-MOF*

FNR/FDH@Zr-MOF was defined as the spatiotemporal encapsulation of tandem enzymes with order of FNR first and then *Cb*FDH. Generally, 1 mg of activated Zr-MOF was added into FNR solution (1.0 mL), which was furhter incubated at 4 ^o^C for 12 h. The nanoparticle was centrifuged and washed with ultrapure water for three times to get FNR@Zr-MOF. And then, *Cb*FDH solution (1.0 mL) was added and incubated at 4 ^o^C for another 12 h. The nanoparticle was centrifuged and washed with ultrapure water for three times to get FNR/FDH@Zr-MOF.

**Measurements**

**1. The** **CbFDH activity assay**

The activity assay of *Cb*FDH was carried out according to the previous reported literature.^2, 3^ In general, to a PBS solution (5.0 mL, 0.5 M, pH=7.4) including 2-hydroxy-1-ethanethiol (0.1 M), *Cb*FDH solution (0.5 mL), NAD^+^ solution (0.5 mL, 16.7 mM), and sodium formate (0.5 mL, 1.67 M) were added. The mixture was further reacted in a water bath at 37 ^o^C for 1 h. UV-vis measurement was employed for detecting the absorption of reduced NADH at 340 nm. The concentration of the *Cb*FDH was measured *via* BCA assay with a standard BSA curve. The enzymatic activity of *Cb*FDH was defined as the mole value of generated NADH catalyzed by the unit weight of *Cb*FDH at unit time with the equation:

$$U\left( Cb\mathrm{FDH} \right)=\frac{A_{340 nm}\left( \mathrm{NADH} \right)\cdot V}{\varepsilon\cdot l\cdot m_{Cb\mathrm{FDH}}\cdot t}(mmol \cdot\mathrm{mg}^{-1}\cdot h^{-1})$$

in which *A*_340 nm_ and *ε* were the absorption value and the molar extinction coefficient (6.22×10^3^ L·mol^-1^·cm^-1^) of NADH at 340 nm*.* The *V*, *l, t* and *m*_CdFDH_ were the volume of solution (mL), optical distance (cm), catalytic reaction time (h) and weight of *Cb*FDH (mg), respectively. The activity of CbFDH we used was measured to be 31.8 μmol·h^-1^·mg^-1^, which was comparable with the literature results.

**2. Stability of FDH@Zr-MOF in harsh conditions**

For the stability test, the encapsulated FDH@Zr-MOF and free *Cb*FDH samples were exposed to elevated temperatures (50 °C) for 60 min, different organic solvents (DCM and ACN) at 4 °C, and trypsin solution (0.5 mg∙mL^−1^) for 60 min, respectively. After being washed with fresh buffer, the enzyme activity was carried out using reported activity assay (in Section 1). The relative activity of free FDH and FDH@Zr-MOF samples under different harsh conditions were recorded *versus* the activity of free FDH at pH 7.4 (which was defined as 100%).

**3. The degradation of Zr-MOF and release of enzymes**

The degradation of Zr-MOF was carried out according to the modified literatures.^4^ Generally, FDH/FNR@Zr-MOF was incubated into K_3_PO_4_ solution (20 mM, pH=7.0) overnight to totally dissolve the MOF structures. The supernatants was collected to measure the enzyme encapsulation by SDS-PAGE and BCA assays.

**4. Thermogravimetric analyses (TGA) measurements**

Thermogravimetric analyses (TGA) were performed on a TA Q500 analyzer (TA Instruments, USA). The pretreated samples were heated from 60 to 650 °C at a rate of 10 °C∙min^-1^ under N_2_ flowing.

**5. Confocal laser scanning microscopy (CLSM) measurements**

A ZEISS LSM 900 equipped with Airyscan 2 was used to observe the colocalization of Zr-MOF hybrids. Generally, *Cb*FDH and FNR enzymes were covalently labelled by FITC and CSC with green, and blue fluorescence emission, respectively. CLSM was used to verify the encapsulation behavior of both enzymes within Zr-MOF (red fluorescence).

**6. Solid UV-visible diffuse reflection spectroscopy (UV-vis DRS)**

Solid ultraviolet visible diffuse reflectance (UV-vis DRS) spectroscopy can be used to calculate the band gap width of the material, and can be used to study the light absorption performance of the catalyst in photocatalysis. UV-2600i ultraviolet visible spectrophotometer equipped with an integrating sphere for testing, using BaSO_4_ as a reference.

The band gap width is calculated using the *Tauc* plot method:

$${(\alpha hv)}^{\frac{1}{n}}=A(hv-E_{g})$$

In which $\alpha, h, v, A$ are the absorption coefficient, Planck constant, incident photon frequency, respectively and a constant. In the equation, *n* is related to the type of semiconductor (n=0.5 for direct bandgap, n=2 for indirect bandgap). $E_{g}$ value is the band gap width of semiconductor.

**7. Electrochemical and photocurrent measurements**

The cyclic voltammetry (CV), Mott-Schottky curve (MS), and the photocurrent response (I-T) of samples were carried out using the Chenhua CHI 760E electrochemical workstation equipped with a standard three-electrode system. A platinum mesh (1×1 cm^2^) electrode and a Ag/AgCl/KCl (3.5 M) electrode as the counter electrode and reference electrode, respectively, and 0.5 M Na_2_SO_4_ (60 mL) was used as electrolyte. 1 × 2 cm^2^ of conductive glass (FTO) was used as working electrode, and ultrasonically cleaned with ethanol and water for three times before use. 1.0 mg of sample was dispersed in 950 μL of anhydrous ethanol and 50 μL Nafion solution, and ultrasound for 10 min. The ethanol solution of the sample was dropped onto the FTO glass and dried under an infrared heating drying lamp. For photocurrent measurement, a 300 W Xenon lamp equipped with a UV cut-off filter (λ > 420 nm) was used as visible light source.

**8. Photocatalytic regeneration of NADH**

The concentration of photogenerated NADH is detected by detecting an absorption change of 340 nm using the UV-Vis absorption spectrum. The final concentration of NAD^+^ and NADH were calibrated using the Lambert-Beer law according to their own molar extinction coefficient (1.72×10^4^ L·mol^-1^·cm^-1^ at 260 nm for NAD^+^, and 6.22×10^3^ L·mol^-1^·cm^-1^ at 340 nm for NADH). The photogenerated NADH was quantitatively analyzed by preparing a standard NADH solution and drawing a standard curve.

The photocatalytic regeneration of NADH from NAD^+^ was carried out in a quartz reactor at room temperature. The photocatalytic regeneration of NADH was conducted in 5 mL Tris-buffer (0.5 M, pH=7.4) containing NAD^+^ (1.0 mM) as cofactor, ascorbic acid (AA, 200 mM) as sacrificial agent, and different photocatalysts (5.0 mg). A 300 W xenon lamp (93 mW∙cm^-2^) equipped with a 420 nm cutoff filter was used as a light source (CEL-PF300-T8, CEAULIGHT, Beijing, China). The reaction system was first incubated under dark conditions for 30 min to balance adsorption and desorption, and then was exposed to light. At each time point, 100 μL of the testing medium was sampled and centrifuged, followed by tenfold dilution prior to measurement. The photoregenerated NADH was quantitatively calculated through the NADH standard curve at 340 nm.

**9. NADH production *via*** **alternatively light on/off cycles**

The testing sample was made according to the aforementioned method. A xenon lamp equipped with a 420 nm cutoff filter was used as a light source. After balance, the reaction system was first exposed to the Xe light (93 mW∙cm^-2^) for 30 min. 100 μL of the testing medium was sampled and centrifuged, followed by tenfold dilution prior to UV-vis measurement, which was light “on” for the first cycle. After that, a light screen was used for 30 min between light source and reaction cell to block the optical path. The same sampling procedure was used prior to UV-vis measurement, the NADH yield was measured as the light “off” for the first cycle. Additional cycles were executed by adhering to the aforementioned cyclical steps.

**10. The artificial photosynthesis of formic acid from CO_2_**

The photosynthesis of formic acid from CO_2_ was performed within the quartz cuvette reactor at room temperature, using a 300 W xenon lamp (93 mW∙cm^-2^) with a 420 nm cutoff filter as light source. Generally, 5.0 mL tris-buffer (0.5 M, pH=7.4) containing NAD^+^ (1.0 mM) as cofactor, ascorbic acid (AA, 200 mM) as sacrificial agent, and different photocatalysts (5.0 mg) was used as reaction mixture, and was bubbled with CO_2_ gas for 0.5 h before use. At time intervals, 400 µL of sample was taken out and mixed with 50 µL of DSS (5.0 mM) and 50 µL of D_2_O for measuring formic acid using ^1^H NMR measurement using DSS (final concentration 0.5 mM) as internal standard. The final concentration of formic acid was determined using the following equation:

$$\boldsymbol{C}=\frac{5}{4}\times\boldsymbol{N}_{H, DSS}\cdot\boldsymbol{C}_{\mathrm{DSS}}\cdot\frac{\boldsymbol{A}_{\delta=7.9}+\boldsymbol{A}_{\delta=8.4}}{\boldsymbol{A}_{\delta=0}}$$

In which $\boldsymbol{C}$ is the concentration of photofixed CO_2_ which is existed in formic acid and formate in solution. $\boldsymbol{N}_{H, DSS}$ and $\boldsymbol{C}_{\mathrm{DSS}}$ are the number of hydrogen atom in DSS molecule (*N* = 9) and concentration of DSS in solution (0.5 mM), respectively. $\boldsymbol{A}_{\delta=0}$, $\boldsymbol{A}_{\delta=7.9}$, and $\boldsymbol{A}_{\delta=8.4}$ are the integral area at δ = 0 for DSS, δ = 7.9 for HCOO^-^, and δ = 8.4 for HCOOH, respectively.

**11. The Apprent Quantum Yield Measurement (AQY)**

The measurement of apparent quantum yield (AQY) for NADH evolution was as follows: the photocatalytic NADH regeneration was conducted in 5 mL Tris-buffer (0.5 M, pH = 7.4) containing NAD^+^ (1.0 mM), ascorbic acid (AA, 200 mM), and photocatalysts (5.0 mg). A 300 W xenon lamp equipped with a 420 nm bandpass filters was used as a light source (CEL-PF300-T8, CEAULIGHT, Beijing, China). The AQE was calculated according to the follow Equation:

$$AQY\left( \% \right)=\frac{2\times n\times N_{A}\times h\times c}{S\times P\times t\times\lambda}\times100\%$$

Where, n is the molar amount of generated NADH, N_A_, h and c are the Avogadro constant (6.022×10^23^ mol^-1^), the Planck constant (6.626×10^-34^ J·s), and the light speed (3×10^8^ m·s^-1^), respectively. S, P, t, and λ are the irradiation area (28.26×10^-4^ m^2^), the power density (W·m^-2^), the irradiation time (s) and the wavelength of monochromatic light (m), respectively.

**Results and Characterization**


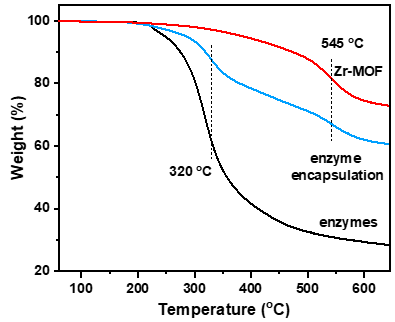


**Figure S13.** Thermogravimetric analysis of Zr-MOF crystals, enzymes and after enzyme encapsulation.


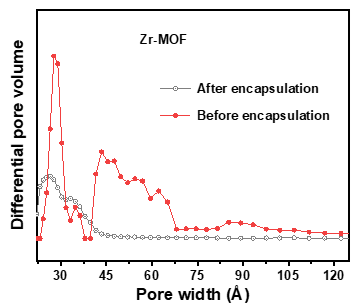


**Figure S14.** Pore size distribution of Zr-MOF before and after enzyme encapsulation.


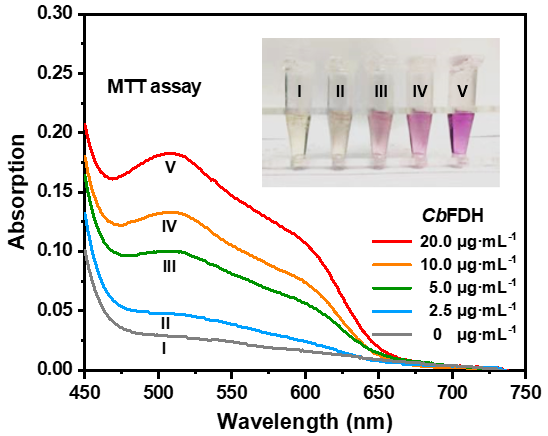


**Figure S15.** UV-vis spectra of MTT reduced by NADH generated in situ from *Cb*FDH catalysis (at 0, 2.5, 5.0, 10.0 and 20.0 μg∙mL^-1^) in the presence of NAD^+^.

**
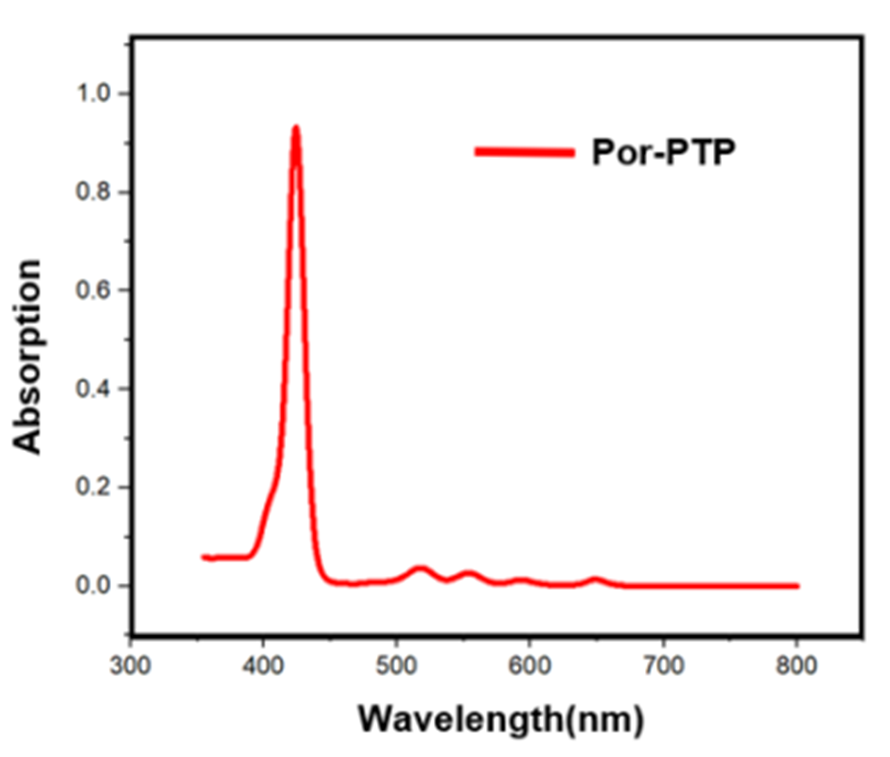
**

**Figure S16.** The UV-vis spectra of Por-PTP.


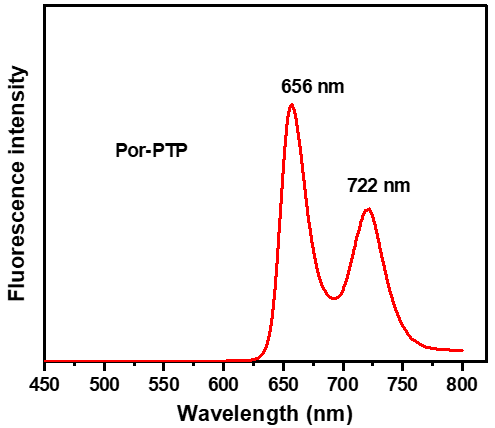


**Figure S17.** The PL emission of Por-PTP ligand.


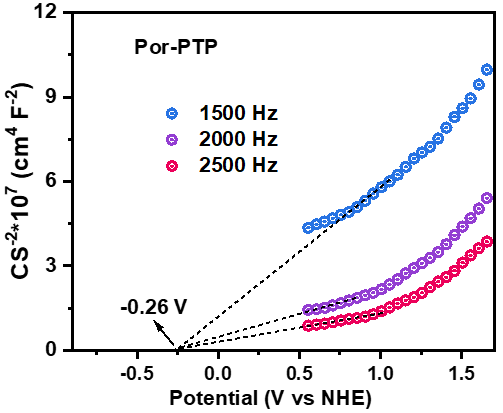


**Figure S18.** Mott-Schottky (M−S) profile of Por-PTP.


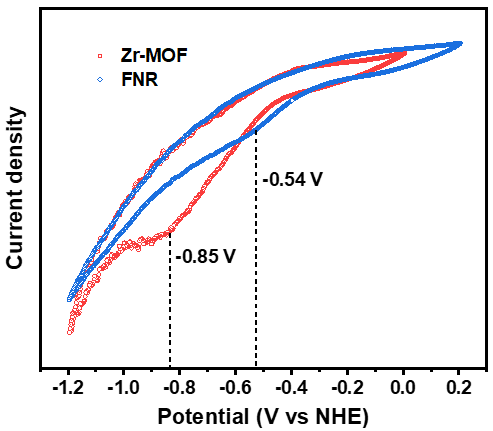


**Figure S19.** The CV spectra of FNR (blue) and Zr-MOF (red).


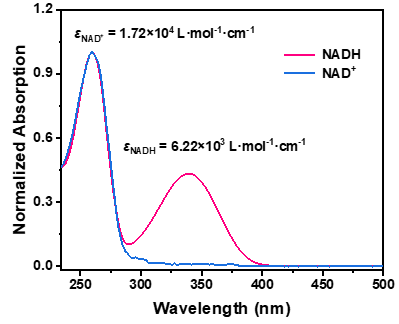


**Figure S20.** UV-vis spectra of NAD+ and NADH.


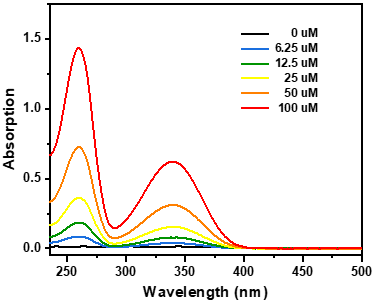


**Figure S21.** UV-vis spectra of NADH at 0, 6.25, 12.5, 25, 50, and 100 uM.


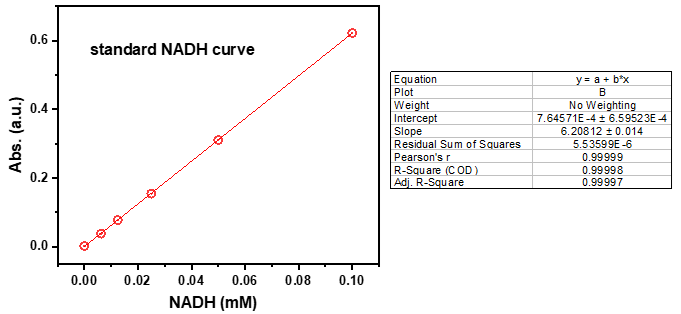


**Figure S22**. Standard curve of NADH absorption at 340 nm *versus* concentration at 0, 0.00625,0.0125, 0.025, 0.05, and 0.1 mM.


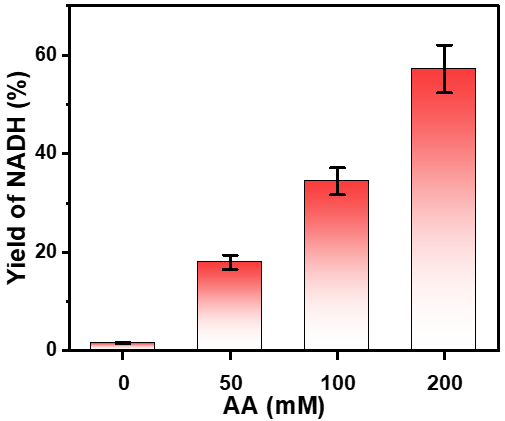


**Figure S23.** Photocatalytic generation of NADH over FNR+FDH@Zr-MOF under different AA concentration. (PD=93 mW∙cm^-1^, [FNR]=25 ug∙mL^-2^). PD refers to power density.


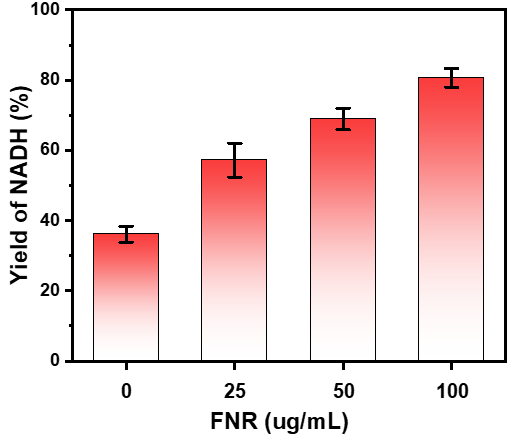


**Figure S24**. Photocatalytic generation of NADH over FNR+FDH@Zr-MOF under different FNR concentration. (PD=93 mW∙cm^-2^, [AA]=200 mM).


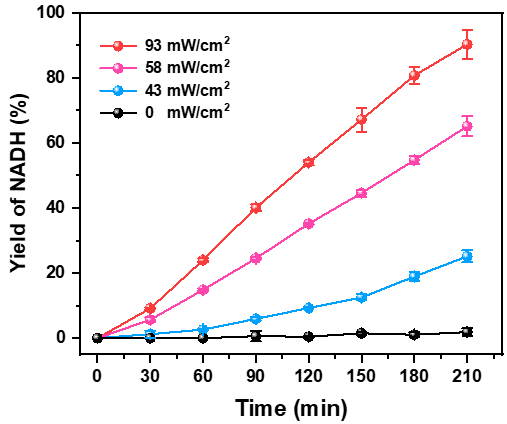


**Figure S25.** Photocatalytic generation of NADH over FNR+FDH@Zr-MOF under light irradiation with different power densities. ([AA]=200 mM, [FNR]=100 ug∙mL^-1^).

**Table S1**. NADH production and HCOOH conversion rate for FDH immobilized with different materials.

| **Samples** | **Light** | **NADH**  **Production** | **HCOOH**  **Content** | **Conversion Rate**  **(****μmol/g/h)** | **Refs.** |
| --- | --- | --- | --- | --- | --- |
| FDH/FNR@Zr-MOF(5 mg) | >420 nm | 85 % (2 h) | 55 mM (12 h) | 4580 | This work |
| FDH@Rh-NU-1006 (1.8 mg)^a^ | 400-700 nm | 28% (2 h) | 144 mM (24 h) | 3300 | ^5^ |
| FDH@RhCp*-NKCOF-113 (10 mg) | >420 nm | 80% (1 h) | 420 μg (2.5 h) | 365 | ^3^ |
| FDH+TCPP/SiO2/Rh HNPs (0.4 mg)^b^ | >420 nm | 75%（3 h） | 100 μmol (4 h) | 62500 | ^6^ |
| CCGCMAQSP (0.5 mg) | >420 nm | 46% (2 h) | 111 μmol (2 h) | 111000 | ^7^ |
| FDH+0.6% PDI/CN (10 mg) | AM 1.5 | 75%（3 h） | 114.8 μmol (9 h) | 1276 | ^8^ |
| FDH@Rh-H_2_TCPP-UiO-66-NH_2_ (1.0 mg) | >400 nm | 61% (1.5 h) | 244 μg/mL (4 h) | 1326 | ^9^ |
| FDH@MAF-7,TPE-C_3_N_4_/PEI/Rh (10.5 mg) | >420 nm | 28% (30 min) | 16.75 mM (9 h) | 532 | ^10^ |
| FDH@Rh_m3_-N-PCN (12 mg) | = 450 nm | 65.6% (20 min) | 5.0 mM (12 h) | 104 | ^11^ |
| In-CdS@ZIF-8&FDH (3 mg) | >420 nm | 94.1% (4 h) | 267.18 μM (24 h) | 3.7 | ^12^ |
| PS I&NADPH-FDH (0.1 mg) | <420 nm | / | 47 μM (2.5 h) | 19 | ^13^ |

Note: ^a^ The mass refers to the content of Pyrene-based ligand, and the catalytic conversion rate of formic acid is measured based on this.

^b^The mass refers to the content of TCPP, and the catalytic conversion rate of formic acid is measured based on this.

**References**

(1) Wang, T. C.; Bury, W.; Gómez-Gualdrón, D. A.; Vermeulen, N. A.; Mondloch, J. E.; Deria, P.; Zhang, K.; Moghadam, P. Z.; Sarjeant, A. A.; Snurr, R. Q.; et al. Ultrahigh Surface Area Zirconium MOFs and Insights into the Applicability of the BET Theory. *J. Am. Chem. Soc.* **2015**, *137* (10), 3585-3591.

(2) Lam, E.; Miller, M.; Linley, S.; Manuel, R. R.; Pereira, I. A. C.; Reisner, E. Comproportionation of CO_2_ and Cellulose to Formate Using a Floating Semiconductor-Enzyme Photoreforming Catalyst. *Angew. Chem. Int. Ed.* **2023**, *62* (20), e202215894.

(3) Zhao, Z.; Zheng, D.; Guo, M.; Yu, J.; Zhang, S.; Zhang, Z.; Chen, Y. Engineering Olefin-Linked Covalent Organic Frameworks for Photoenzymatic Reduction of CO_2_. *Angew. Chem. Int. Ed.* **2022**, *61* (12), e202200261.

(4) Chen, Y.; Li, P.; Modica, J. A.; Drout, R. J.; Farha, O. K. Acid-Resistant Mesoporous Metal–Organic Framework toward Oral Insulin Delivery: Protein Encapsulation, Protection, and Release. *J. Am. Chem. Soc.* **2018**, *140* (17), 5678-5681.

(5) Chen, Y. J.; Li, P.; Zhou, J. W.; Buru, C. T.; Dordevic, L.; Li, P. H.; Zhang, X.; Cetin, M. M.; Stoddart, J. F.; Stupp, S. I.; et al. Integration of Enzymes and Photosensitizers in a Hierarchical Mesoporous Metal-Organic Framework for Light-Driven CO_2_ Reduction. *J. Am. Chem. Soc.* **2020**, *142* (4), 1768-1773.

(6) Ji, X.; Wang, J.; Mei, L.; Tao, W.; Barrett, A.; Su, Z.; Wang, S.; Ma, G.; Shi, J.; Zhang, S. Porphyrin/SiO_2_/Cp*Rh(bpy)Cl Hybrid Nanoparticles Mimicking Chloroplast with Enhanced Electronic Energy Transfer for Biocatalyzed Artificial Photosynthesis. *Adv. Funct. Mater.* **2018**, *28* (9), 1705083.

(7) Yadav, R. K.; Baeg, J.-O.; Oh, G. H.; Park, N.-J.; Kong, K.-j.; Kim, J.; Hwang, D. W.; Biswas, S. K. A Photocatalyst–Enzyme Coupled Artificial Photosynthesis System for Solar Energy in Production of Formic Acid from CO_2_. *J. Am. Chem. Soc.* **2012**, *134* (28), 11455-11461.

(8) Zhang, P.; Hu, J.; Shen, Y.; Yang, X.; Qu, J.; Du, F.; Sun, W.; Li, C. M. Photoenzymatic Catalytic Cascade System of a Pyromellitic Diimide/g-C_3_N_4_ Heterojunction to Efficiently Regenerate NADH for Highly Selective CO_2_ Reduction toward Formic Acid. *ACS Appl. Mater. Interfaces* **2021**, *13* (39), 46650–46658.

(9) Xing, X.; Liu, Y.; Lin, R.-D.; Zhang, Y.; Wu, Z.-L.; Yu, X.-Q.; Li, K.; Wang, N. Development of an Integrated System for Highly Selective Photoenzymatic Synthesis of Formic Acid from CO_2_. *ChemSusChem* **2023**, *16* (5), e202201956.

(10) Tian, Y.; Zhou, Y.; Zong, Y.; Li, J.; Yang, N.; Zhang, M.; Guo, Z.; Song, H. Construction of Functionally Compartmental Inorganic Photocatalyst–Enzyme System via Imitating Chloroplast for Efficient Photoreduction of CO_2_ to Formic Acid. *ACS Appl. Mater. Interfaces* **2020**, *12* (31), 34795-34805.

(11) Zhang, Y.; Liu, J. Bioinspired Photocatalytic NADH Regeneration by Covalently Metalated Carbon Nitride for Enhanced CO_2_ Reduction. *Chem. Eur. J.* **2022**, *28* (55), e202201430.

(12) Zhou, J.; Tian, X.; Yu, S.; Zhao, Z.; Ji, Y.; Schwanebrg, U.; Chen, B.; Tan, T.; Cui, Z.; Wang, M. In-CdS@ZIF-8&FDH photo-enzyme nanosystem with high NADH regeneration ability via indium doping for enhanced CO_2_ reduction to formic acid. *Chem. Eng. Sci.* **2024**, *285*, 119613.

(13) Ihara, M.; Kawano, Y.; Urano, M.; Okabe, A. Light Driven CO_2_ Fixation by Using Cyanobacterial Photosystem I and NADPH-Dependent Formate Dehydrogenase. *PLOS ONE* **2013**, *8* (8), e71581.
